# ﻿Characterization of the mitochondrial genomes for *Ophiostomaips* and related taxa from various geographic origins and related species: large intron-rich genomes and complex intron arrangements

**DOI:** 10.3897/imafungus.16.159349

**Published:** 2025-07-22

**Authors:** Jigeesha Mukhopadhyay, Alvan Wai, B. Franz Lang, Georg Hausner

**Affiliations:** 1 Department of Microbiology, University of Manitoba, Winnipeg, MB, R3T 2N2, Canada University of Manitoba Winnipeg Canada; 2 Robert Cedergren Centre for Bioinformatics and Génomiques, Département de Biochimie et Médecine Moléculaire, Université de Montréal, Montréal, Quebec H3C 3J7, Canada Université de Montréal Montréal Canada

**Keywords:** Bluestain fungi, complex introns, group I and group II introns, mitogenomes, phylogenetics

## Abstract

The *Ophiostomatales* are of economic concern, as many are blue-stain fungi and some are plant pathogens. The mitogenomes of members assigned to this order exhibit size polymorphism despite having highly conserved gene order, owing to the variable number of introns and intron insertion sites. In this work, eleven blue-stain fungi, including nine strains of *Ophiostomaips* with a varied distribution across North America and New Zealand, were sequenced and compared with other members of the *Ophiostomatales*. A pan-mitogenome intron landscape has been prepared to demonstrate the distribution of the mobile genetic elements and to provide insight into the evolutionary dynamics of introns among members of this group of fungi. The size variation among these mitogenomes (from about 23.8 kb to 152 kb) shows high correlation to the presence and absence of introns. Examples of complex or nested introns composed of two or three intron modules have been observed in some *O.ips* strains. RNA-seq data suggests possible splicing pathways with regard to resolving these complex introns. Mitochondrial DNA and RNA data for *O.ips* provides the basis for future studies relating to gene annotation, alternative splicing, evolutionary intron dynamics, and taxonomic investigations for members of the *Ophiostomatales*.

## ﻿Introduction

Species assigned to the *Ophiostomatales* are characterized by producing ascocarps with asci that are randomly produced at the base of the ascocarp; the asci are short-lived and deliquesce with the ascospores being released as a sticky droplet at the tip of the perithecial necks (Hausner et al. 1993a; Zipfel et al. 2006). Many of these features, along with tall conidiophores, are usually associated with adaptations towards insect dispersal. The taxonomy of the *Ophiostomatales* has undergone many revisions (Zipfel et al. 2006; De Beer et al. 2022). Historically, the order accommodated fungi that are currently placed into two families, *Ceratocystidaceae* and *Ophiostomataceae*. DNA sequences revealed that convergent evolution of morphological characters generated conflicting taxonomic schemes for these fungi (Hausner et al. 1993a, b, c; Réblová et al. 2011). Currently, *Ceratocystidaceae* is assigned to the *Microascales* and *Ophiostomataceae* to the *Ophiostomatales* (Hausner et al. 1993c; De Beer et al. 2016); based on molecular data, 16 genera are currently accepted for inclusion in the *Ophiostomatales*; however, there could be up to 24 lineages/genera based on a recent analysis using four nuclear markers (Zipfel et al. 2006; De Beer et al. 2022). *Ophiostomaips* (Rumbold, Nannfeldt) is an economically important bark beetle-associated blue-stain (sapstain) fungus (Seifert 1993) that has a global distribution and appears to be vectored by a broad range of insects, including *Ips* and *Dendroctonus* species (Rumbold 1931; Rane et al. 1987; Uzunovic et al. 1999; Wingfield et al. 2017; Wang et al. 2019). *O.ips* produces pleomorphic asexual states and is morphologically similar to *O.montium* and *O.adjuncti* (Upadhyay 1981). Hence, its taxonomy, based on culture and morphological characteristics, can be challenging (Kim et al. 2003).

Mitochondria are semi-autonomous organelles present almost universally among eukaryotes. They contain genetic material, known as mitochondrial DNA (mtDNA) or mitogenome. Mitochondrial processes are supported by *trans*-acting nuclear genes (reviewed in Zardoya 2020). Mitochondrial DNA for filamentous members of the *Ascomycota* shows size polymorphism despite encoding a similar set of core genes. These genes include those encoding for components of the respiratory chain, which include the cytochrome oxidase subunits (*cox1*, *cox2*, and *cox3*), the cytochrome b (*cob*), subunits of NADH dehydrogenase (*nad1* to *nad6*, including *nad4L*), along with *rnl* and *rns* (large and small subunit RNAs involved in protein translation), and components of ATP synthase (*atp6*, *atp8*, *atp9*), and a set of tRNA genes. Sometimes, the ribosomal protein S3 gene (*rps3*) can be encoded within an *rnl* intron or be found as a free-standing gene (reviewed in Hausner 2003; Wai et al. 2019).

Fungal mitogenomes harbor mobile genetic elements such as group I and group II introns, composed of a ribozyme component and, in some instances, an open reading frame (ORF) that encodes an intron-encoded protein (IEP). IEPs can catalyze intron mobility and, in many instances, promote intron splicing (Belfort et al. 2002; Lambowitz and Zimmerly 2011; Mukhopadhyay and Hausner 2021). The splicing mode and the corresponding RNA folds differentiate group I from group II introns (Michel and Dujon 1983; Belfort et al. 2002). For group I introns, IEPs tend to be site-specific homing endonucleases (HEs), and for group II introns, reverse transcriptases (RTs). These IEPs have applications in biotechnology, such as genome editing reagents and/or possible regulatory switches to control gene expression (Hafez and Hausner 2012; Stoddard 2014; Guha et al. 2017; Belfort and Lambowitz 2019; Gomes et al. 2024). In fungal mitogenomes, two families of HEs can be recognized based on the presence of conserved amino acid motifs: the LAGLIDADG or GIY-YIG families of HEs (Stoddard 2014). Homing endonuclease genes (HEGs) can be free-standing or embedded within group I intron sequences. HEGs can move independently from their ribozyme partners (Mota and Collins 1988), but it has been suggested that intron-encoded HEGs tend to co-evolve with their ribozyme partners (Megarioti and Kouvelis 2020). Finally, there are a few instances where group II introns encode HEGs (Toor and Zimmerly 2002; Zimmerly and Semper 2015).

In some instances, mobile introns can invade an existing intron and form a nested or complex intron arrangement. Originally described as twintrons (two modules), the position of the internal intron module within the pre-existing (resident) intron could determine whether its splicing is essential for the splicing of the resident intron (Copertino and Hall 1994). It was assumed that the internal intron module splices first before the resident intron module can generate a splicing-competent RNA fold (reviewed in Hafez and Hausner 2015). The name ‘zombie’ twintrons (half-dead, half-alive) was suggested by Zumkeller et al. (2020) for cases where splicing of external introns does not depend on prior splicing of internal introns and the nested intron arrangement can splice out as one unit.

Complex introns have been identified in the mitogenomes of various fungi, including members of the *Ophiostomatales* (Hafez et al. 2013; Guha and Hausner 2014; Guha et al. 2018; Zubaer et al. 2021; Mukhopadhyay et al. 2023). In some examples, the splicing of the internal intron module will reconstitute the ORF that it interrupted, thereby allowing for its expression (Guha and Hausner 2014). Other complex introns have the potential to generate splicing intermediates whereby the internally located intron ORF gets fused to the upstream exon (Guha et al. 2018; Zubaer et al. 2021). The latter might be an example of splicing-mediated ‘core-creep’ (Zubaer et al. 2021). It has been reported by Edgell et al. (2011) that many intron-encoded ORF sequences are fused, in frame, with the upstream exon of their host gene, and this might enhance the expression of the IEP.

This study examines the mitogenomes from nine *O.ips* (or *O.ips*-like) strains isolated from North America and New Zealand. The sequences were compared to available sequences from members of the *Ophiostomatales*, including the closely related species *O.montium* and *O.adjuncti*. This study is part of the long-term project to generate and apply mitogenomes for members of the *Ophiostomatales* as a resource for taxonomic applications, plus to gain a better understanding of the evolutionary dynamics of introns and mitogenome diversity.

## ﻿Materials and methods

### ﻿Source of culture, culturing methods, and extraction of nucleic acids

Fungal strains used in the study are listed in Table 1. Seven strains of *O.ips*: WIN(M) 1478, WIN(M) 1480, WIN(M) 1481, WIN(M) 1486, WIN(M) 1487, WIN(M) 1488, and WIN(M) 1001, and two strains morphologically resembling *O.ips*, *Ophiostoma* sp. WIN(M)1515 and *O.adjuncti*WIN(M) 502 were maintained on malt extract agar (MEA, supplemented with yeast extract; 30 g/L malt extract, 1 g/L yeast extract, 20 g/L agar) slants and agar plates (~40 mL MEA). Cultures were incubated in the dark at 20 °C for up to 2 weeks.

For the extraction of nucleic acids, three 250 mL Erlenmeyer flasks containing 80 mL of PYG + ME broth (1 g/L peptone, 1 g/L yeast extract, 2 g/L D-glucose, and 3 g/L malt extract) were each inoculated with ten agar blocks (2 mm × 2 mm × 1 mm) and incubated in the dark at 20 °C for up to 10 days. Fungal mycelium was harvested from liquid media by vacuum filtration through a Whatman® Grade 1 filter paper in a Büchner funnel. Mycelium was ground up in a pre-chilled mortar with a pestle and acid-washed sand, and whole genome DNA for next-generation sequencing was recovered and purified as previously described (Wai and Hausner 2021). DNA samples (30 ng/μL DNA in a final volume of 100 μL) were sent to MicrobesNG (Units 1–2 First Floor, The BioHub, Birmingham Research Park, 97 Vincent Drive, Birmingham, B15 2SQ, United Kingdom) for whole genome Illumina sequencing (Illumina NovaSeq 6000 (Illumina, San Diego, USA) using a 250 bp paired-end protocol).

For two strains of *O.ips* [WIN(M) 1478 and 1480], RNA was prepared from 7–10-day-old cultures (~50 mg wet weight of fungal mycelium) using the RNAeasy Plant Mini Kit (Qiagen, Valencia, CA, USA) following the protocol outlined by the manufacturer.

Contaminating DNA was removed from the nucleic acid samples using the TURBO-DNase kit (Applied Biosystems, ABI, Foster City, CA, USA). RNA was prepared according to specifications in the User Guide for Illumina Sequencing Technologies by Genome Quebec and submitted for preparing total RNA libraries without poly(A) selection, rRNA/tRNA depletion, or size selection. RNA samples for RNA-seq analysis were prepared in duplicate for two strains of *O.ips*, and the entire RNA-seq analysis was carried out twice with RNA samples prepared in 2021 and 2022. The bulk RNA sequence reads for WIN(M) 1478 (including trimmed, corrected, and concatenated FASTQ files) can be accessed under the BioProject PRJNA1143047, SRA accession SRX25593433.

**Table 1.** Eight strains of *Ophiostomaips* and *ips*-like and one strain of *O.adjuncti*, which were sequenced through NGS and analyzed for this study, are tabulated.

No.	Species/Strain	GenBank ID	Culture Collection	Source/Notes
1	*Ophiostomaips*WIN(M) 1478	PQ043840	TB 3.11 = UAMH12572	TB, Canada
2	*O.ips*WIN(M) 1480	PQ043832	TB 3.10 = UAMH12573	TB, Canada
3	*O.ips*WIN(M) 1481	PQ043835	TB 2.5 = UAMH12574	TB, Canada
4	*O.ips*WIN(M) 1486	PQ043836	ATCC24285 = UAMH12575	Canada
5	*O.ips*WIN(M) 1487	PQ043837	CBS137.36 = UAMH12579	AUT strain; Oregon, USA
6	*O.ips*WIN(M) 1488	PQ043838	SYPT1-USA-B Colette Breuil = UAMH12576	Washington State, USA
7	*O.ips*WIN(M) 1001	PQ043834	JR 88-130A’ = UAMH12577	New Zealand
8	*Ophiostoma* sp. WIN(M) 1515	PQ043839	JR 82-83a = UAMH12578	New Zealand
9	*O.adjuncti*WIN(M) 502	PQ043833	ATCC34942 = UAMH12571	Ex-type; New Mexico, USA

WIN(M) = University of Manitoba; TB = Thunderbay, Canada; UAMH = UAMH Centre for Global Microfungal Biodiversity, Toronto, ON, Canada; ATCC = American Type Culture Collection; CBS = Westerdijk Fungal Biodiversity Institute; JR = James Reid; AUT = authoritative strain.

### ﻿Assembly, analyses, and annotation of mitogenome data

Initial analyses of the Illumina reads were performed on the online platform Galaxy (https://usegalaxy.eu/; Afgan et al. 2018). The reads from MicrobesNG were assessed using FastQC v0.11.9.1. Assemblies were generated by using two programs: SPAdes vs. 3.14.0 (setting the “--careful” option and assembly graph option) and an independent A5-miseq pipeline (with “--end” option set to “5”; Tritt et al. 2012; Coil et al. 2015). The program Bandage (Wick et al. 2015) was used to examine the assembly graph files generated from SPAdes to aid in the recovery of potential mitogenome contigs. To more efficiently recover mitogenome-derived reads and assemblies, GetOrganelle (Jin et al. 2020) v1.7.5, with the organelle type set to fungus mitogenome (i.e., -F fungus_mt), was applied. Additional mitogenomes, *O.montium* (SRR19396179), *O.ips* (SRR19396180), and other members of the *Ophiostomatales* were obtained from GenBank and the Sequence Read Archive (SRA; Shumway et al. 2010; Leinonen et al. 2011). The collection, processing, and analyses of SRA data for mitogenomes were previously described in Wai and Hausner (2021) and Mukhopadhyay et al. (2023).

The mtDNA contigs were annotated using the MFannot program (Lang et al. 2023), setting “Genetic Code” to 4 (mold), and RNAweasel (Prince et al. 2022) for predicting tRNAs and group I and group II introns. Predictions of tRNAs were also performed with tRNAscan-SE 2.0 (Chan and Lowe 2019). Annotations of protein-coding genes (PCGs) (*atp6*, *atp8*, *atp9*, *cob*, *cox1–3*, *nad1–6*, and *nad4L*) and noncoding genes (i.e., *rnl* and *rns* and tRNAs) were verified by comparative sequence analysis from data obtained from GenBank (Benson et al. 2013).

Sequence alignments were generated for all protein-coding genes with corresponding genes from *Tolypocladiuminflatum* serving as a reference genome for naming introns [based on the position of insertion; Zhang and Zhang (2019)]. The rRNA coding genes were compared with those (16S and 21S RNA) of *Escherichiacoli* with regard to intron annotations/naming as described by Johansen and Haugen (2001). Gene annotations were refined with Artemis (Rutherford et al. 2000), and the mitogenomes were visualized using Circos (Krzywinski et al. 2009). Circos was set up with the appropriate coordinates to highlight exon/intron configurations for conserved protein-coding genes and noncoding genes and to plot GC content. The GC plot was generated using a window size of 100 bp and a step size of 20 bp.

### ﻿Reverse transcriptase PCR and RNA sequencing

For one-step RT-PCR, the SuperScript IV One-Step RT-PCR kit (Invitrogen, Carlsbad, CA, USA) was used. *O.ipscob*-490 and *cox3*-640 RT-PCR primers were used (Suppl. material 1: tables S1, S2) for amplification, followed by verification of cDNA using agarose gel electrophoresis on 1% gel.

The sequencing of RNA libraries (cDNA) was performed on an Illumina NovaSeq 6000 platform (PE100 4 X 25 million reads/lane). For quality control, obtained paired-end reads were run through FastQC for quality control and Trimmomatic (Bolger et al. 2014) to remove low-quality bases and adapters. Additionally, Rcorrector (Song and Florea 2015; custom script courtesy of F. Lang, University of Montreal) was used to error-correct Illumina reads (i.e., correct for RT artifacts and random sequencing errors).

### ﻿RNA-seq reads mapping and visualization

The annotated mitogenomes for strains WIN(M) 1478 and 1480 were used for reference-based mapping of the RNA-seq data; however, *de novo* assembly of the transcriptome was also performed using rnaSPAdes (Bushmanova et al. 2019). Two independent RNA-seq runs were performed using these two strains. The reads were aligned to the reference genome using STAR (https://github.com/alexdobin/STAR; Dobin et al. 2013). The output from STAR, a BAM file, was used for visualizing mapped reads with the Integrative Genomics Viewer, IGV (http://www.broadinstitute.org/igv; Robinson et al. 2011). IGV also provided validation for the prediction of exonic and intronic regions for all genes (i.e., gene structure prediction from MFannot). On IGV, soft-clipped bases (bases in the 5′ and 3′ ends of the read that are not contiguous in the alignment with the reference sequence) were viewed to facilitate detection of transcript structural variations that might arise due to non-canonical splicing (such as alternative splicing events).

### ﻿Phylogenetic analysis of mitochondrial protein-coding regions

A data set comprising 65 mitogenomes (including the nine mitogenomes studied in this paper) was generated that included two members of the *Eurotiales* and several non-*Ophiostomatales* fungi. This data was composed of 13 concatenated amino acid sequences derived from the following protein-coding genes: *atp6*, *atp8*, *cob*, *cox1–3*, *nad1–6*, and *nad4L*. As some members of the *Ophiostomatales* do not encode *atp9*, this sequence was not included in the dataset (Wai and Hausner 2021; Zubaer et al. 2021). The data were aligned using MAFFT version 7 (Katoh and Standley 2013), and the alignment was manually adjusted with AliView version 1.25 (Larsson 2014). The alignment was analyzed with MrBayes 3.2.7a (Huelsenbeck and Ronquist 2001) for inferring phylogenetic trees. A fixed-rate amino acid substitution model was estimated with the mixed model function implemented in MrBayes (MB). Rate variation among sites was modeled with a combination of the invariable sites model and gamma model (i.e., rates from a gamma distribution). Other parameters were left at their default values. The MB analysis was performed with 2,000,000 generations with a sampling frequency of 1,000. During this analysis, the cpREV model was estimated to have the highest probability. The first set of 25% of sampled trees was discarded (burn-in), and the remaining trees were used to estimate posterior probabilities and to construct the 50% majority rule consensus tree. The aligned data set was also analyzed applying maximum likelihood (ML) as implemented in IQTREE2 (Minh et al. 2020). The LG model (+I+F) was applied (based on the best-fitting model prediction by the Model option in MEGA X1 (Tamura et al. 2021)), and 1,000 bootstrap replicates were analyzed to estimate node support values. Trees obtained from MEGA X1, MrBayes, and IQ-TREE2 were visualized and annotated with the FigTree program v1.4.4 (http://tree.bio.ed.ac.uk/software/figtree/), and members of the *Eurotiales* were selected as the outgroup.

## ﻿Results

### ﻿The mitogenomes: size variation and gene content

**Figure 1.**
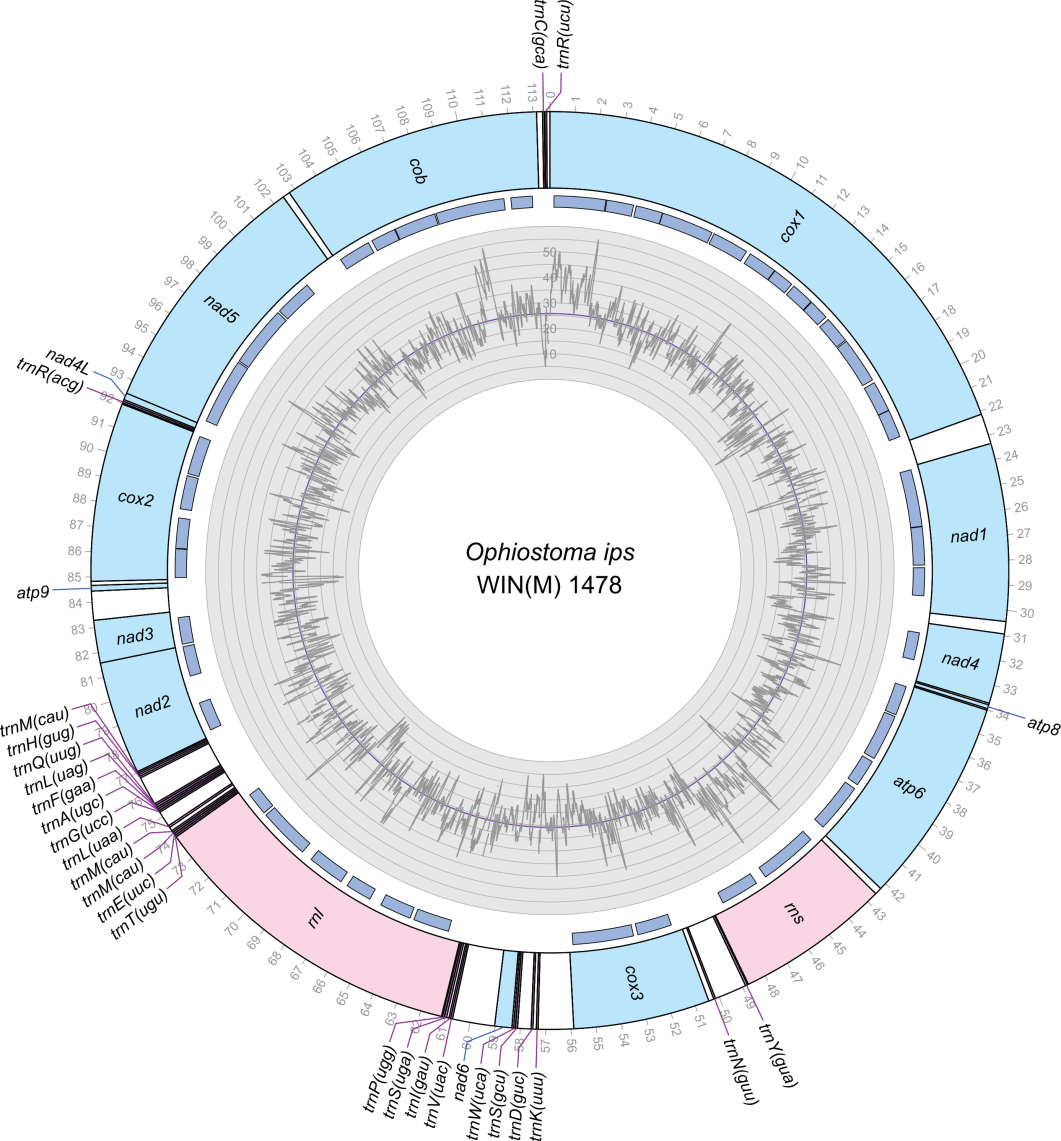
Circular representation of the mitochondrial genome of *O.ips*WIN(M) 1478. Genes, introns, and GC plots are shown on the outer, middle, and inner tracks, respectively. The purple line of the GC plot corresponds to the average GC content of the mitochondrial genomes. The tick marks on the outer track label every 1,000^th^ nucleotide, starting from the beginning of the *cox1* gene. All labeled genes are encoded on the same strand.

**Figure 2.**
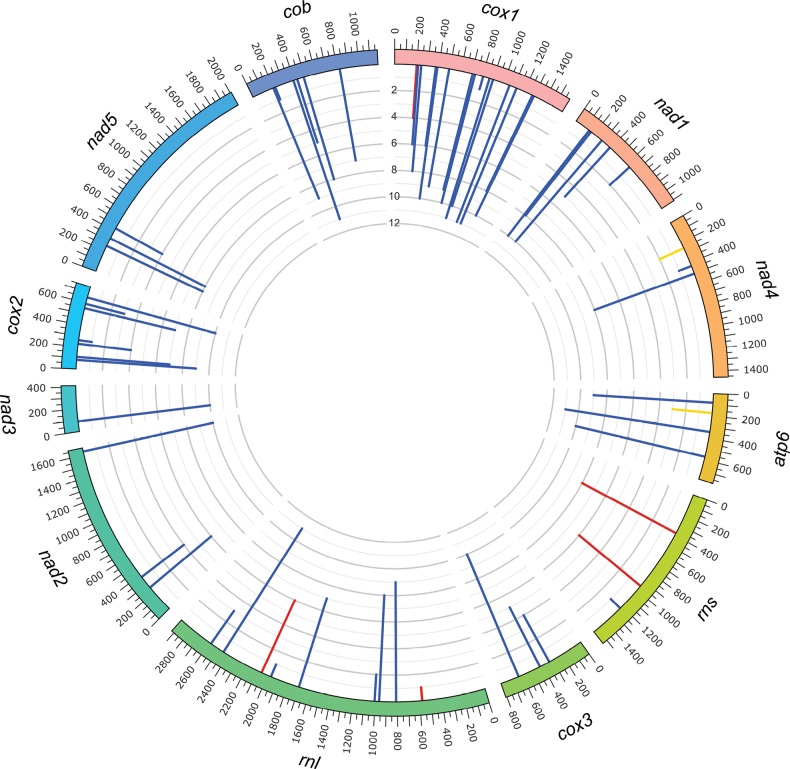
The pangenomic intron landscape for the studied members of the *Ophiostomatales*. The landscape was generated by Circos and shows all intron insertion sites and their frequencies.

Previously, Zubaer et al. (2021) analyzed the scaffold corresponding to the mitogenome of *O.ips*CBS 138721 (GenBank accession number: NTMB01000349.1). However, this draft mitogenome was not complete (the *atp8* region was missing, and *nad4* and *atp6* were incomplete). Therefore, in this study, additional strains were examined from two different geographic regions to get a better representation of the mitogenome for this economically important species. A representative mitogenome for *O.ips* [WIN(M) 1478] is presented in Fig. 1. The remaining mitogenomes characterized in this study are presented as circular annotated molecules in Suppl. material 1: figs S1–S7; for GenBank accession numbers, see Table 1. The pan-mitogenomic intron landscape for the studied mitogenomes [*O.ips* strains WIN(M) 1478, WIN(M) 1480, WIN(M) 1481, WIN(M) 1486, WIN(M) 1487, WIN(M) 1488, WIN(M) 1001, WIN(M) 1515, *O.montium* SRR19396179, *O.ips* SRR19396180, and *O.adjuncti*WIN(M) 502] is presented in Fig. 2.

The mitogenomes studied vary in size, with WIN(M) 1478 being the largest at 113,671 bp and WIN(M) 1515 being the smallest at 39,957 bp. The mitogenome sizes for the rest are as follows: WIN(M) 1481 at 111,826 bp, WIN(M) 1486 at 80,045 bp, WIN(M) 1487 at 85,284 bp, WIN(M) 1488 at 106,900 bp, *O.adjuncti*WIN(M) 502 at 112,641 bp, and WIN(M) 1001 at 104,698 bp. Additionally, we included *O.montium* SRR19396179 (76,883 bp) and *O.ips* NTMB01000349 (97,849 bp). The annotated mitogenome sequences for *O.montium* (SRR19396179) and *O.ips* (SRR19396180) can be accessed in GenBank as third-party submissions: BK068991 and BK068990, respectively.

All mitogenomes studied encode the ribosomal RNA genes *rns* and *rnl* and the mitochondrial core set of 14 protein-coding genes (*atp6*, *atp8*, *atp9*, *cob*, *cox1–3*, *nad1–6*, and *nad4L*). As observed in other members of the *Ophiostomatales* (and members of the *Sordariomycetes*), the ribosomal protein RPS3 is encoded within a group IA-type intron embedded within the *rnl* gene (*rnl* position mL2450 reviewed in Wai et al. 2019).

All nine mitogenomes sequenced in this study and the additional *O.ips* and *O.montium* mitogenomes encode a set of tRNAs that cover all 20 standard amino acids. The tRNA genes are arranged into a few interspersed clusters, with the majority of tRNA genes arranged upstream and downstream of the *rnl* (largest cluster between *rnl* and *nad2*) gene. Smaller groupings of tRNA genes are located between the *cox3*/*nad6* and the *nad6*/*rnl* genes. The tRNA configuration is the same for all the studied *O.ips* and related mitogenomes, with *trnM* as the last member of the largest cluster downstream of the *rnl* gene. Each of the studied mitogenomes has three copies of *trnM* (CAU), two copies with alternate versions for *trnL* (UAG and UAA), two copies with alternative versions of *trnR* (ACG and UCU), and two copies with alternative versions of *trnS* (GCU and UGA). A putative tRNA, *trnP* (CCU), is detected in the *nad4*-257 intron of *O.adjuncti*WIN(M) 502.

Mauve progressive alignment of the studied *O.ips* and related mitogenomes shows that, despite the size polymorphism among the strains of the same species, the genomes are collinear and show no evidence of rearrangements (Suppl. material 1: fig. S8). The gene order and orientation are all identical: *cox1*, *nad1*, *nad4*, *atp8*, *atp6*, *rns*, *cox3*, *nad6*, *rnl*, *nad2*, *nad3*, *atp9*, *cox2*, *nad4L*, *nad5*, and *cob* (Suppl. material 1: fig. S9). Other features noted are the *nad2* and *nad3* genes being contiguous and the overlap between the *nad4L* and *nad5* ORFs by one nucleotide, i.e., the last nucleotide of the *nad4L* stop codon serves as the first nucleotide of the *nad5*ORF. These gene arrangements have been observed in other members of the *Ophiostomatales* and *Ascomycota* (Abboud et al. 2018; Mukhopadhyay et al. 2023).

### ﻿The mitogenome intron component

The *cox1* gene has the most intron insertion sites [13 in WIN(M) 1478, 1480, 1481, 1488, and 1001; 11 in WIN(M) 1486; 9 in WIN(M) 1487; and 5 in WIN(M) 1515], followed by the *rnl* gene [6 in WIN(M) 1478, 1480, 1481, and 1001; 4 in WIN(M) 1488; 3 in WIN(M) 1486 and 1487; and 1 in WIN(M) 1515] and the *cob* gene [5 in WIN(M) 1478, 1480, 1481, and 1488; 4 in WIN(M) 1001 and WIN(M) 1486; and 2 in WIN(M) 1487]. For *O.adjuncti*WIN(M) 502, 12 intron insertion sites are noted in *cox1*, 6 in *rnl*, and 2 in *cob*.

**Figure 3.**
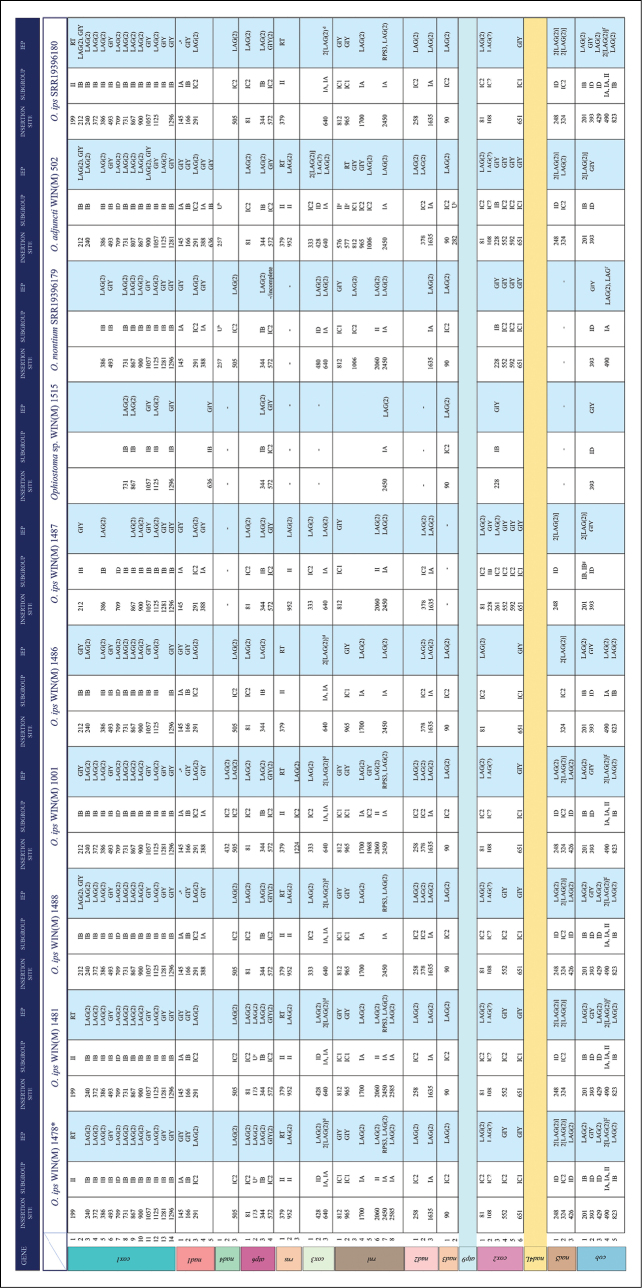
Summary of introns and intron-encoded proteins observed within the mitochondrial genomes of the studied members of the *Ophiostomatales*. Intron insertion sites for protein-coding genes were based on *Tolypocladiuminflatum* (NC 036382.1; Zhang and Zhang 2019). No introns have been identified for *nad6* and *atp8* genes. Intron subgrouping applies only to group I introns; group II introns are annotated as “II”; unidentifiable = no identifiable group I or II intron based on MFannot/RNAweasel results and manual inspection. Intron-encoded protein (IEP) associated with intron insertion site based on BLASTx results: LAG(2), double-motif LAGLIDADG homing endonuclease; RT, reverse transcriptase; GIY, GIY-YIG homing endonuclease; “–“, no conserved motif detected based on BLASTx results. a. Does not appear to encode ORF (GIY); b. The beginning of the intron resembles ending of nad4 gene; for WIN(M) 502, encodes additional nucleotides in comparison to *Ophiostomamontium* (SRR19396179) with *trnR* (ccu) accounting for ˜71 bp of the “extra” ˜1781 bp [LAG (could be single/double-motif; note: this is in addition to LAG(2) shared by both]; c. For *atp6* -173, and *nad3* -282, there is partial imperfect duplication of the downstream exon at the beginning of the intron; it is difficult to identify the intron type; *atp6* -173 encodes *trnI* (aau) in WIN(M) 1478, 1480, and 1481; d. Complex twintron *cox3* -640 consisting of two group IA intron modules in tandem; e. Two group II introns at positions *rnl*-576 and *rnl*-577 are separated by a mini-exon (A) in an arrangement based on domain V; one RT-ORF encoded by *rnl* -577; f. Complex trintron consisting of a group II intron module between two gr IA modules; for *O.montium* (SRR19396179), *cob*490 (IA) encodes LAG(2) that appears to be interrupted by potentially single-motif LAG; g. *cob*-201 complex intron with two modules of group IB introns (according to MFannot) and encoding two double-motif LAGLIDADG endonucleases.

The mitogenomes of WIN(M) 1478, 1480, and 1001 contain 46 introns, while the mitogenomes of WIN(M) 1481 and 1488 contain 45 introns. *O.ips* SRR19396180, with a smaller genome at 96,039 bp, contains 39 introns, whereas WIN(M) 1487, at 85,284 bp, contains 33 introns, and WIN(M) 1486, at 80,045 bp, contains 32 introns. *Ophiostomaadjuncti*WIN(M) 502 contains 45 introns, whereas *O.montium* SRR19396179 contains 30 introns. The smallest of the studied mitogenomes, the *O.ips*-like WIN(M) 1515, contains only 12 introns. Fig. 3 summarizes the location of all introns, their classification, and types of intron-encoded ORFs. Group I introns encode either GIY-YIG or LAGLIDADG type ORFs, with the *nad1*-145 group I introns in WIN(M) 1488 and 1001 showing no evidence of an ORF. For *atp6*-173 in WIN(M) 1478, 1480, and 1481, it was difficult to identify the intron type, which had a partial imperfect duplication of the downstream exon at the beginning. Among the seven sequenced *O.ips* strains, WIN(M) 1487 differs from the remaining studied *O.ips* and related species in this study in terms of intron content. WIN(M) 1515, originally identified as *O.ips*, is now referred to as *Ophiostoma* sp. WIN(M) 1515 or *ips*-like in this paper, as there are large differences in mtDNA size and intron content. The complex *cob* and *cox3* introns are also absent in WIN(M) 1487 and WIN(M) 1515. In addition, strain WIN(M) 1515 has a much smaller genome at 39,957 bp compared to the other *O.ips* strains. Mitogenome sizes correspond to intron numbers, and this is exemplified by WIN(M) 1515 with 12 introns and *Ophiostomapiceae* at 33,599 bp with 6 introns compared with WIN(M) 1478 (=1480) at 113,671 bp and 46 introns.

Most of the observed introns belong to group I, while group II introns are mostly found in the ribosomal genes (*rnl*, *rns*) and in the *cox1* and *cob* genes of some members. Group I introns are observed at three insertion sites in *atp6*, five in *cob*, fourteen in *cox1*, six in *cox2*, three in *cox3*, five in *nad1*, three in *nad2*, one in *nad3*, two in *nad4*, three in *nad5*, seven in *rnl*, and one in *rns.* Conversely, group II introns have been identified only at *cob*-490 (as part of a complex intron), *cox1*-199, three positions in *rnl* (576, 577, and 2060), and two positions in *rns* (379 and 952).

The group I introns usually encode for double- or single-motif LAGLIDADG and GIY ORFs. Group I introns with complex ORF arrangements are described as follows: the *atp6*-572 intron encoded two GIY-YIG-ORFs in all members, except WIN(M) 1515 and WIN(M) 1487, which encoded only one GIY-YIG-ORF. The *cox1*-212 intron, in addition to the GIY-YIG-ORF encoded by WIN(M) 1001, 1486, and 1487, encodes a double-motif LAGLIDADG ORF in WIN(M) 1488. Similarly, *nad5*-248 in WIN(M) 1478, 1480, and 1481 encodes two double-motif LAGLIDADG ORFs, whereas in WIN(M) 1001, 1487, and 1488, only one double-motif LAGLIDADG ORF is encoded. The *rps3* gene is encoded within the mL2450 intron in all members of the *Ophiostomatales*. However, in strains WIN(M) 1478, 1480, 1481, and 1001, the *rps3* sequence is part of a gene fusion where the C-terminal rps3 coding segment is fused in-frame with a double-motif LAGLIDADG coding region, as previously observed by Sethuraman et al. (2009) in some members of the *Ophiostomatales*.

All the *cox1* group II introns encode RTs. In contrast, group II introns in the ribosomal genes mostly encode homing endonucleases; *rns*-952 in WIN(M) 502, 1478, 1480, 1481, 1487, 1488, and *rnl*-2060 in WIN(M) 1478, 1480, 1487, 1481, and 1001 encode double-motif LAGLIDADG-type proteins. Group II introns are observed as components of complex intron arrangements in *cob*-490 in the following strains: WIN(M) 1001, 1478, 1480, 1481, 1488, and *O.ips* SRR19396180. In WIN(M) 502, two group II introns appeared to form a tandem twintron-like arrangement at the *rnl*-576 position; however, a one-nucleotide exon could be identified that indicates group II introns inserted at positions *rnl*-576 and *rnl*-577 (see next section for more details).

### ﻿Complex intron arrangements

In this study, two complex/nested intron arrangements are observed in the *cob* (cytochrome b; *cob*-490) and *cox*3 (cytochrome c oxidase subunit III; *cox3*-640) genes in the following *O.ips* strains: WIN(M) 1478, 1480, 1481, 1488, and 1001. Of these, the complex *cox3*-640 is also noted in WIN(M) 1486, but a single group IA module was observed at the *cob*490 position. Both complex arrangements have been reported in *O.ips* (NTMB01000349.1; CBS138721), and their RNA folds have been described in Zubaer et al. (2021). A schematic overview of these complex introns (*cob*-490 and *cox3*-640) can be found in Zubaer et al. (2021).

**Figure 4.**
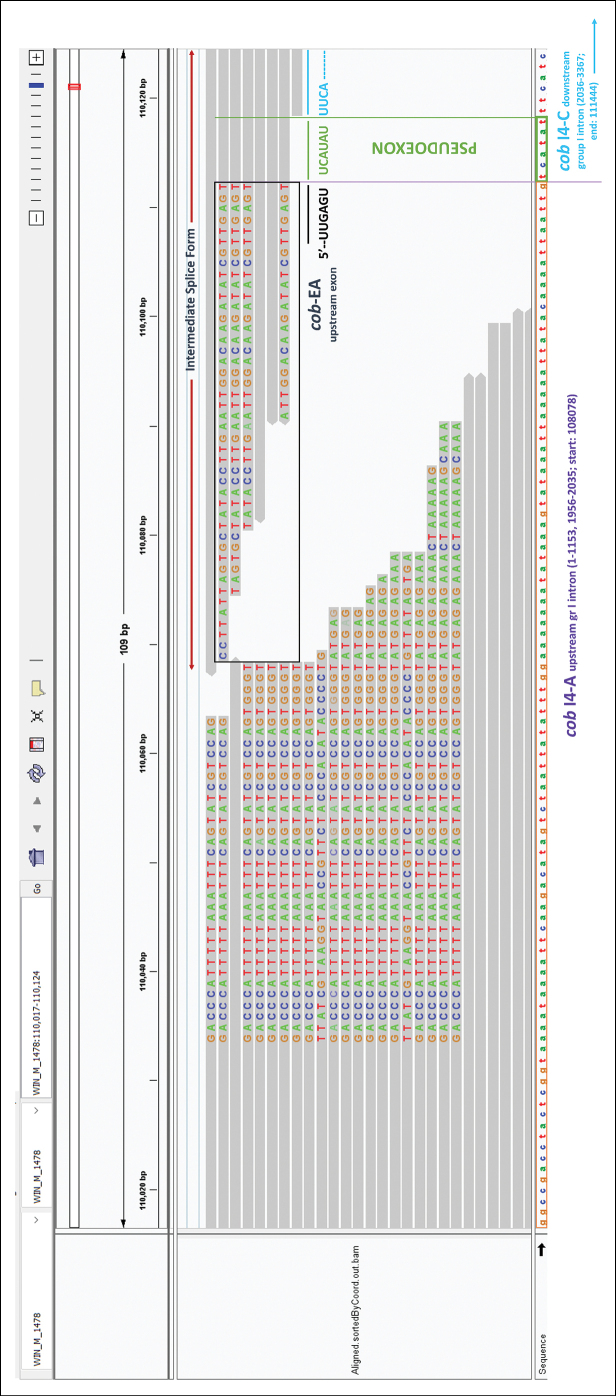
RNA-Seq reads mapped to the annotated mitogenome of WIN(M) 1478, showing the “pseudoexon” sequence. The IGV snapshot shows the mapped RNA-Seq reads, with the following highlighted: the upstream intron sequence of *cob*-490 (relative to *T.inflatum*) in purple (*cob* I4-A; group I intron with start position 108078 relative to the mitogenome sequence; spanning from 1-1153 and 19562035 relative to the ungapped I4 intron sequence); the downstream intron sequence of *cob*-490 in blue (*cob* I4-C; group I intron with end position 111444 relative to the mitogenome sequence; spanning from 2036-3367 relative to the ungapped I4 intron sequence) containing the pseudoexon sequence (TCATAT) in green (spanning from 2036-2041 relative to the ungapped I4 intron sequence). The numbers in brackets represent the positional range of each intron element, taking the start of *cob* I4 as position 1.

Briefly, the *cox3*-640 complex intron appears to be composed of two group IA1 intron modules in a tandem arrangement with the upstream and downstream group IA1 intron modules separated by an inter-intron module sequence. RNA-seq data appears to show that this complex intron splices as one unit, as splicing intermediates suggesting otherwise were not recovered. The *cob*-490 complex intron consists of three distinct modules, and each module appears to contain all the necessary components for splicing (Zubaer et al. 2021). The three modules are a group IA1 intron that is interrupted by an ORF-less group IIB intron module, and this composite element is inserted within the P1 loop of a group IA1 intron module (presumably the resident intron). The group II intron appears to be located within the P8 loop of its host group I intron module. Both group I intron components contain ORFs that encode double-motif LAGLIDADG-type homing endonucleases. There is a short sequence separating the two group I intron modules. This inter-intron module sequence is speculated to act as a “pseudoexon” because this sequence would allow the upstream group I intron component to splice out by utilizing the pseudoexon for the formation of the P10 helix (fig. 10 in Zubaer et al. 2021). This is supported by the RNAseq data presented in this study (Fig. 4), which suggests that the “upstream” intron is spliced out prior to the “downstream” intron module. RNA-seq reads were mapped to the reference annotated genomes of WIN(M) 1478 and 1480 and visualized on IGV. This visualization shows evidence for transcript reads in which the pseudoexon sequence ‘TCATAT’ was fused to the upstream exon sequence (the last 8 nt on the 3′ end being ‘CGTTGAGT’), which represents the “intermediate splicing step” according to the ratchet model (also referred to as recursive splicing, reviewed in Hafez and Hausner 2015), where the upstream intron has been spliced in the first step leading to this fusion (Fig. 4). Two independent runs of RNA sequencing yielded reproducible reads of this sequence fusion, indicating that they are not likely sequencing artifacts, supporting the proposed model that this complex intron could use ratchet splicing as a mode of RNA processing.

RNASeq reads show the “intermediate splice form” as hypothesized in the “ratchet” splicing model from Zubaer et al. (2021) has been shown where the *cob*-EA upstream exon sequence is fused to the pseudoexon sequence (part of *cob* I4-C), indicating that for these mapped reads, the upstream intron *cob* I4-A has been spliced out in a preliminary splicing step. This map validates the ratchet splicing model of the complex intron, where intron modules utilize the pseudoexon sequence for splicing in multiple steps through intermediate splice forms.

**Figure 5.**
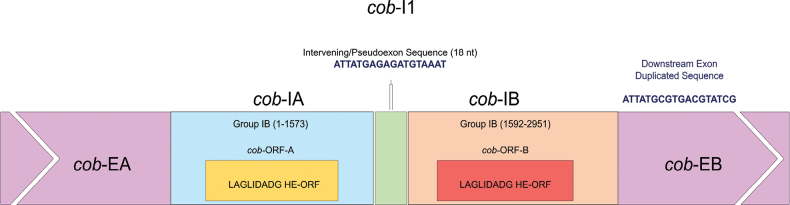
A schematic diagram of the *cob*-201 intron in *O.ips*WIN(M) 1487. *cob*-I1, the entire complex twintron at *cob*-201 position (relative to *T.inflatum*). *cob*-IA is the upstream group IB intron; *cob*-EA, the upstream exon; *cob*-ORF-A, encoded by *cob*-IA; *cob*-IB is the downstream group IB intron; *cob*-ORF-B, encoded by *cob*-IB; and *cob*-EB, the downstream exon. LAGLIDADG represents the type of homing endonuclease ORF encoded by the two group IB intron modules. The numbers in brackets represent the positional range of each intron element, taking the start of *cob*-I1 as position 1. The two group I introns are separated by an intervening sequence of length 18 nt, which can be interpreted as a “pseudoexon” that seems to be duplicated (partially) as a downstream exon sequence. This tandemly arranged twintron can also be interpreted as two independent group IB intron modules at positions 201 and 219, respectively (relative to *T.inflatum*).

In *O.ips*WIN(M) 1487, the *cob*-201 intron appears to be a complex intron composed of two group IB intron modules arranged in a tandem configuration; both modules encode LAGLIDADG ORFs (Fig. 5). However, the second intron module at the 5′-end contains a short 18 nt sequence that could be a duplication of the downstream exon; this could lead to an alternate interpretation whereby the two group IB introns are separated by an 18 nt exon. The “intron” 18 nt component potentially encodes the amino acid sequence that is expected for the downstream exon. What further complicates the interpretation is that the intronic version of the 18 nt resembles the exonic version of this sequence in *O.ips*WIN(M) 1478 and related strains, and the WIN(M) 1487 downstream exonic 18 nt sequence resembles the corresponding sequence as found in *O.adjuncti*WIN(M) 502.

**Figure 6.**
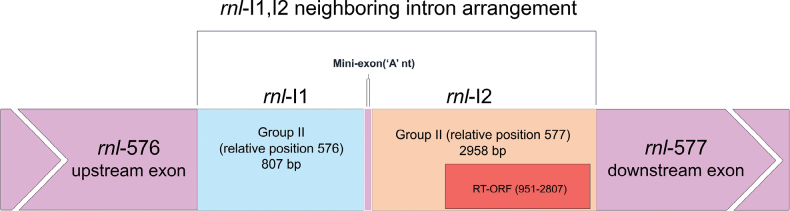
A schematic diagram of the neighboring arrangement of the *rnl*-576 and 577 introns in *O.adjuncti*WIN(M) 502. *cob*-I1, the entire intron arrangement starting at the *rnl*-576 position (relative to *E.coli*). *rnl* -I1 is the first group II intron at position 576; *cob*-ORF-A, encoded by *cob*-IA; *rnl* -I2 is the second group II intron at position 577; RT-ORF, encoded by *rnl*-I2, and the range is given taking the first nucleotide of the intron as position 1; the upstream and downstream exons are also shown. The two group II introns are separated by a single nucleotide ‘A,’ which can be called a “mini-exon”.

**Figure 7.**
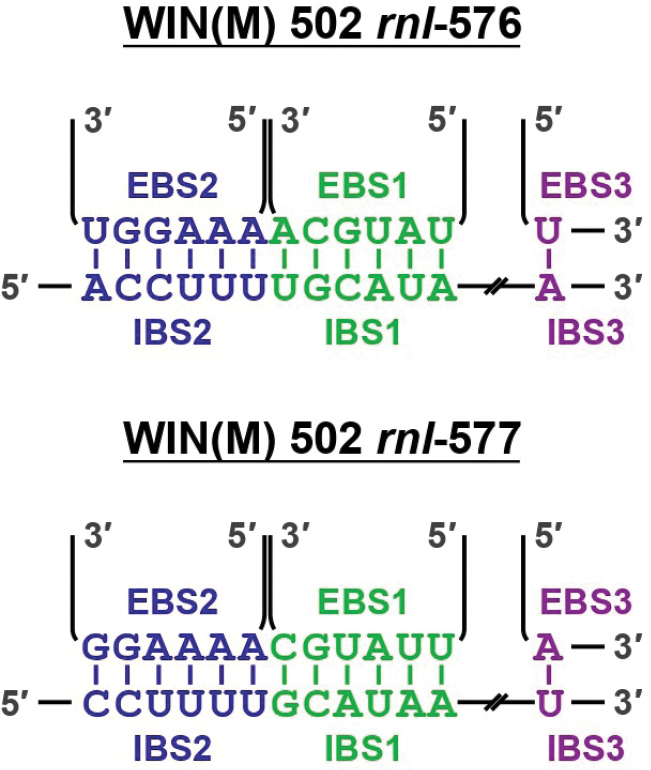
IBS-EBS interactions of WIN(M) 502 *rnl*-576 and *rnl* -577. Possible interactions are shown at the folded secondary structure levels, based on domain V identified through RNAweasel.

An interesting intron arrangement has also been identified in *Ophiostomaadjuncti*WIN(M) 502 (schematically represented in Fig. 6). Here, two group II introns have been predicted at positions *rnl*-576 and *rnl*-577. Possible interactions between intron- and exon-binding sequences (IBS1, 2, and 3 and EBS1, 2, and 3, respectively) at the folded secondary structure levels for the two introns are shown in Fig. 7 (for a detailed view of intron folds, see Suppl. material 1: figs S10, S11). The intron at *rnl*-576 does not start or end with typical, conserved nucleotides (begins with ‘UUGCG’ and ends with ‘GGU’ instead of the conserved ‘GUGYG’ and ends with ‘AY’). Though not as common, ‘UUGCG’ has been reported previously (Michel et al. 1989). The non-canonical ending is favored by the predicted IBS3/EBS3 interactions and the RNA folds for two group II introns that exhibit all the expected domains (Fig. 7). The *rnl*577 group II intron appears to encode an RT-ORF (in domain IV), while the *rnl*-576 intron is ORF-less. These two intron modules appear to be separated by one nucleotide exon. Both introns, based on structural features, are group IIB types. The EBS3/IBS3 interactions and the ability to model DVI of the upstream group II and the presence of the conserved beginning (‘GUGYG’) and DI of the downstream group II. [i.e., upstream group II DVI is immediately followed by ‘GGU’ (instead of the conserved ‘RAY’), then A (exon), then the downstream group II that starts with the conserved ‘GUGCG-A-DI’] favor the existence of the one-nucleotide exon at this position; a similar configuration was previously reported in Mayers et al. (2021) for members of the genus *Ceratocystis*.

### ﻿Phylogenetic groupings observed through mitogenome analysis

**Figure 8.**
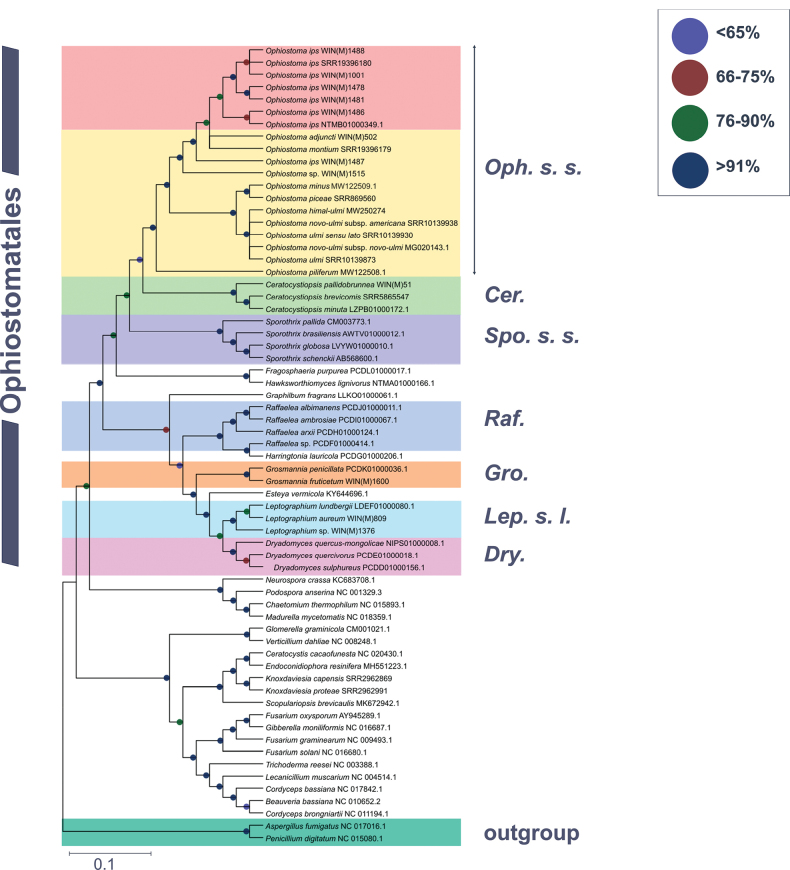
Phylogenetic relationships of 65 fungal species belonging to the *Ascomycota*, including 43 members of the *Ophiostomatales*, are presented, based on concatenated amino acid sequences composed of *atp6*, *atp8*, *cob*, *cox1*, *cox2*, *cox3*, *nad1*, *nad2*, *nad3*, *nad4*, *nad4L*, *nad5*, and *nad6*. *Aspergillusfumigatus* and *Penicilliumdigitatum* are selected as the outgroups. Maximum Likelihood (LG + I + F) as implemented in MEGA XI was used to generate a phylogenetic tree using the bootstrap option (1,000 pseudoreplicates) to estimate node support values. A second tree was constructed based on Bayesian inference, and the posterior probability (PP) support values from the 50% majority Bayesian (MB) consensus tree (Cprev+F+I model) are represented in combination with the maximum likelihood node support values (bootstrap support) on this tree. Branch lengths are proportional to the number of substitutions per site (see scale bar). NCBI/MitoFun accession numbers for each sequence are indicated, and where not available, a strain identifier is used.“*Cer.*” = “*Ceratocystiopsis*”, “*Drya*” = *Dryadomyces* (=*R.sulphurea* complex)”,“*Est.*” = “*Esteya*”, “*Fra.*” = “*Fragosphaeria*”, “ *Gra.*” = “*Graphilbum*”, “*Gro.*” =“*Grosmannia*”, “*Har.*” = “*Harringtonia* gen. *nov.* (=*R.lauricola* complex), “*Haw.*”= “*Hawksworthiomyces*”, “*Lep. s. l.*” = “*Leptographiumsensu lato* ”,“*Oph. s.s.*” = “*Ophiostomasensu stricto*”, “*Raf.*” = “*Raffaelea*”.

The phylogenetic analysis of 65 concatenated mitochondrial protein sequences, including 43 species that belong to the *Ophiostomatales*, yields a topology showing monophyletic groupings for the *Microscales*, *Hypocreales*, *Glomerellales*, *Sordariales*, and *Ophiostomatales* (Fig. 8). Within the *Ophiostomatales*, several monophyletic lineages could be identified representing the following genera: *Ceratocystiopsis*, *Graphilbum*, *Hawksworthiomyces*, *Raffaelea*, *Harringtonia*, *Dryadomyces*, *Leptographium*, *Esteya*, *Ophiostomasensu stricto*, and *Sporothrix* (De Beer et al. 2016, 2022). All members of the *Ophiostomatales* can be derived from one branch with high levels of confidence (100%) based on ML (IQ-TREE2; Suppl. material 1: fig. 12S) and Bayesian inference (MrBayes; Fig. 8) analyses. Mitochondrial sequences show that species historically assigned to the genus *Raffaelea* can be resolved into three distinct genera as proposed by De Beer et al. (2022): *Harringtonia*, *Dryadomyces*, and *Raffaelea* (Fig. 8).

Based on the phylogenetic analysis, we can infer one grouping for all *O.ips* strains that includes WIN(M) 1478 (=1480), WIN(M) 1481, WIN(M) 1486, WIN(M) 1488, WIN(M) 1001, *O.ips* NTMB01000349.1, and *O.ips* SRR19396180. However, *O.ips*WIN(M) 1487 appears to branch prior to a branch that groups *O.adjuncti*WIN(M) 502 with a sequence reported for *O.montium* SRR19396179. *Ophiostoma* sp. WIN(M) 1515 was the most distant member of the “*O. ips*” complex examined in this study (“*O. ips*” complex as defined by De Beer et al. 2022).

## ﻿Discussion

### ﻿Mitogenome organization, size, and intron proliferation

The mitogenomes of the nine strains of *O.ips*, *ips*-like WIN(M) 1515, and two related taxa from various global locations (North America and New Zealand) show size polymorphism that can be attributed to the difference in their content of introns and intronic ORFs, leading to genome expansion. Similar observations have been noted for other members of the *Ophiostomatales* (Zubaer et al. 2018, 2021; Wai and Hausner 2021; Mukhopadhyay et al. 2023). Gene order is highly conserved among the *Ophiostomatales*, with some minor variations observed in terms of the tRNA gene set and the presence or absence of the *atp9* gene, which might be compensated by the presence of a nuclear counterpart (Déquard-Chablat et al. 2011; Sellem et al. 2016; Zubaer et al. 2018; Wai and Hausner 2021). The *atp9* gene was encoded by the mitogenome in all members of the *O.ips* complex (Suppl. material 1: fig. S8).

Among fungi, large-scale genome rearrangements were often accompanied by the accumulation of intergenic and intronic sequences, resulting in the increase in mitogenome size (Zubaer et al. 2018; Ye et al. 2020). The movement of organellar introns is assumed to be site-specific, moving from an intron+ allele to a cognate intron- allele, a process referred to as homing. However, introns can potentially integrate into new locations ectopically by invading a new site (some allele or different gene). Once a mobile intron has been inserted into a new target site, its persistence is assumed to depend on drift (neutral evolution) (Goddard and Burt 1999; Gimble 2000; Gogarten and Hilario 2006). Whether an intron is maintained in a population is dependent on drift and/or selection based on the correlation between the energy burden of housing the intron and possible advantages provided by introns, such as fine-tuning gene regulation (Rudan et al. 2018), functioning as environmental sensors (Belfort 2017), promoting hypovirulence (Baidyaroy et al. 2011), and interference with the effects of fungicides (Cinget and Bélanger 2020). Large-scale intron loss events have been reported in the mitogenomes of lichen fungi (Pogoda et al. 2019) and ectomycorrhizal fungi belonging to the genus *Boletus* (Li et al. 2021). This shows that the dynamics of introns and mitochondrial genome expansion and shrinkage are complex and can vary among members of different lineages or among species within the same genus (reviewed in Mukhopadhyay and Hausner 2021).

### ﻿The intron complement for the *O.ips* lineage

In fungi, mitogenomes show an abundance of group I introns in comparison to group IIs, which contrasts with plant mitogenomes, where group II introns have a wide distribution (reviewed in Mukhopadhyay and Hausner 2021). In this study, we have identified a total of 416 introns across 63 insertion sites in the eleven mitogenomes; out of 416 introns, 32 are group II introns. Among these, five are part of a complex intron arrangement, *cob*-490, in *O.ips*WIN(M) 1001, 1478, 1480, 1486, 1488, and SRR19396180, and two group II introns occupy adjacent insertion sites (*rnl*-576, 577) in *O.adjuncti*WIN(M) 502. Group I introns are observed across 55 insertion sites and group II introns across 5 sites, and three sites are occupied by “unidentified” intron types (*atp6*-173, *nad2*-282, and *nad4*-257). There appears to be bias towards group I introns in the examined mitogenomes. This leads to the speculation that the process of group I intron homing might be more successful in fungal mitogenomes in comparison to retrohoming, as noted for group II introns. Group II introns are present in the ribosomal genes (*rns*, *rnl*) and the N-terminal region of the *cox1* gene, sequences that show high sequence conservation. Some group II introns (typically ORF-less) have been observed to be inserted within group I introns as part of a complex intron (see *cob*-490). Some group II introns encode LAGLIDADG ORFs, such as the mS952 and mL2060 introns (Pfeifer et al. 2012), and this might promote a DNA-based homing strategy that might be more successful in certain situations compared to retrohoming-based pathways.

### ﻿Unusual and complex intron arrangements

Introns expand by gaining additional ORFs (such as HEs) or intron modules (Guha et al. 2018). There are instances of internal (non-fused) ORFs being expressed via alternative splicing (Turk et al. 2013; Wai et al. 2019) that can lead to splicing-mediated ‘core-creep,’ as observed for the *cob*-490 trinton. In some instances, “secondary invaders” can insert in-frame to the resident intron ORF sequences that are already fused (in-frame) to the upstream exon. A possible example of this variation of core creep was observed in a *cox1*-212 complex intron in *Leptographium* sp. WIN(M) 1376, where a LAGLIDADG ORF fused in-frame with a resident GIY-YIG ORF and the upstream exon, probably taking advantage of this fusion for more efficient translation of the intron-encoded ORFs (Mukhopadhyay et al. 2023). Some notable complex intron arrangements uncovered in this study are described in the following sections.

#### ﻿i. The *cox3*-640 twintron

The *cox3*-640 twintron (two group I intron modules in tandem) appears to splice as a composite unit based on RT-PCR results and RNA-seq, and the downstream intron module is presumed to be the original resident (native) intron (Zubaer et al. 2021). The *cox3*-640 intron fits the “zombie” definition [as suggested by Zumkeller et al. (2020)], and this might be an energetically favorable cellular process. Tandem-type complex introns, such as *O.ips cox3*-640, have been observed and described in *Ceratocystiopsisbrevicomis* (PCDN01000199.1) and *Grosmanniapenicillata* (PCDK01000036.1) (see Zubaer et al. 2021 supplementary data).

#### ﻿ii. The *cob*-490 trintron

Evidence generated in this study suggests that the *cob*-490 trinton could be processed by a “ratchet” pathway, where the first intron module is removed in a primary step, generating an intermediate RNA molecule that regenerates suitable sequences for the second intron module to assume a splicing-competent RNA fold, including P1 and P10 interactions that allow for its removal and joining of the flanking exons. One must assume that the splicing is instantaneous, and therefore it is difficult to detect rare splice intermediates, especially in non-model fungal systems that do not lend themselves to synchronized culturing strategies. Based on the IGV visualization of mapped RNA-seq reads and observation of soft-clipped bases, we have found reproducible evidence for transcripts mapping to the pseudoexon sequence ‘TCATAT’ associated with the upstream exonic sequences (the last 8 nt on the 3′-end being ‘CGTTGAGT’) (Fig. 4). This arrangement represents the “intermediate splicing step” from the ratchet model, where the upstream intron has been spliced out, leading to the fusion of the upstream exon with the pseudoexon sequence, which allows the formation of a splice-competent fold for the downstream group I intron.

#### ﻿iii. The *cob*-201 complex intron with two group IB modules

Two group IB modules were identified at the *cob*-201 position in *O.ips*WIN(M) 1487. However, this arrangement of two group IB introns can also be interpreted as two independent intron modules at the positions 201 and 219 (relative to the *cob* sequence in the annotation reference, *T.inflatum*). Assuming a complex intron, the 18 nt “intervening sequence” separating the two intron modules could potentially represent a pseudoexon that is required for the splicing of the two intron modules, and eventually it is removed during the processing of the *cob* transcript. The duplication of this 18 nt sequence could have been introduced by the invading intron module, as homing endonuclease-mediated mobility can also modify flanking sequences due to gene conversion (Belfort et al. 2002).

#### ﻿iv. The *rnl*-576 and *rnl*-577 introns separated by a single-nucleotide exon

Two group II intron modules were identified at *rnl*-576 and *rnl*-577 positions in WIN(M) 502. In this neighboring-introns arrangement, a single ‘A’ nucleotide serves as the exon to separate the two introns, and an RT is encoded by the ORF housed in the second (downstream) intron (*rnl*-577). Based on the conservation of the introns, one potential scenario could be that *rnl*-577 inserted prior to the insertion of the ORF-less *rnl*-576 that might have been inserted into the IBS of *rnl*-577 by reverse splicing. One can speculate that, later in time, the insertion of *rnl*576 interfered with the splicing of *rnl*-577, by which the downstream intron has now become dependent on the splicing of *rnl*-576 prior to its own splicing. This scenario is supported by secondary structure modeling of the intron folds (Fig. 7) that shows that the second intron has still maintained its “native” exon binding sequences (EBS1 and EBS2, which are complementary to the last 12 nucleotides of the 5′-exon); the corresponding intron binding sequences (IBS1 and IBS2) for the second intron are restored after splicing of the first intron. The splicing reaction resembles a ratchet-like arrangement as the first splicing event (*rnl*-576) “generates” the splice site for the second group IIB intron (*rnl*-577). In *rnl*-576 and *rnl*-577, the EBS1 for the *rnl*-577 intron seems to interact with the ‘A’ exon plus the upstream exon, and thus, the second intron seems to have its IBS sequences shifted by one nucleotide compared to the first intron. This would support the existence of the single nucleotide ‘A’ exon.

Previously, an example of two *rnl* group II introns separated by an “A” single nucleotide exon was described in two *Ceratocystis* species, *C.lukuohia* and *C.huliohia*. These two elements, referred to as SPAM 3 and SPAM 7 (Mayers et al. 2021), are possible orthologs of the *rnl*-576 and *rnl*-577 introns observed in *O.adjuncti*WIN(M) 502. The authors proposed a tandem splicing mechanism that is essentially the same as what we propose for the *rnl*-576 and *rnl*-577 introns. Related introns based on our analysis (data not shown) have been observed in *Ophiocordycepssinensis*KP835313.1, *Graphilbumipis-grandicollis* sp. nov.

JADHKH000000000.1, and *Graphilbumrectangulosporium* JADHKI010000081.1, but only the downstream intron *rnl*-577 is present, whereas in *Grosmanniapenicillata* MLJV01000071.1, only the upstream *rnl*-576 can be identified). Both introns appear to be in adjacent *rnl* insertion sites based on compatible IBS/EBS sequence interactions.

Examples of short or single nucleotide exons in mitogenomes are scarce; short 3 bp exons were previously reported in fungi (Xavier et al. 2012), and a single nucleotide exon has been reported in the mtDNA*cox1* gene for a placozoa (Osigus et al. 2017). This could potentially be attributed to misannotation, in part due to standard annotators adopting Hidden Markov Models (HMM) that use training datasets with three nucleotide-based codons, which would not favor prediction of very small exons (Yu et al. 2022; Lang et al. 2023).

### ﻿Phylogenetic analysis of mitogenomes

In this study, we examined mitogenomes for species that can be assigned to the following genera of the *Ophiostomatales*: *Ceratocystiopsis* H.P. Upadhyay & W.B. Kendr., *Graphilbum* H.P. Upadhyay & W.B. Kendr., *Dryadomyces* Gebhradt., *Hawksworthiomyces* Z.W. De Beer et al., *Harringtonia* Z.W. De Beer & Procter., *Esteya* J.Y. Liou et al., *Fragosphaeria* Shear., *Grosmannia* Goid et al., *Leptographium* Lagerb. & Melin, *Raffaelea* Arx & Hennebert, *Sporothrix* Hektoen & C.F. Perkins, and *Ophiostoma* Syd. & P. Syd. These genera include economically important tree pathogens and blue-stain fungi (Hausner et al. 2005; Fisher et al. 2012). Historically, due to overlapping and sometimes conflicting morphological characters, this group of fungi has been challenging with regard to generic assignment and species identification (Upadhyay 1981; Zipfel et al. 2006; De Beer et al. 2022). The tree topology supported the currently proposed generic lineages for members of the *Ophiostomatales*. The tree also showed the monophyly of strains belonging to *O.ips* and taxa related to *O.ips*, such as *O.adjuncti* and *O.montium* (Upadhyay 1981). The data also shows that the *O.ips* complex/lineage shares a common ancestor with other members of *Ophiostomasensu stricto*, such as *O.piceae*, members of the *O.ulmi* species complex, and *O.piliferum*. As more mitogenomes become available, they will be valuable in resolving lineages among the members of the *Ophiostomatales*.

*Ophiostomaips* strains from North America and New Zealand all grouped together with two exceptions, strains WIN(M) 1487 and WIN(M) 1515. With regard to WIN(M) 1515, it was previously noted by J. Reid (personal communications) that the ascospores were larger than that described for *O.ips*, although the conidial states for WIN(M) 1515 appeared to match descriptions for *O.ips*. The mitogenome phylogenetic analysis, along with its significantly smaller mitogenome, appears to suggest it should be referred to as “*ips*-like” *Ophiostoma* sp., and more studies are needed to assign it to a species. However, the position of WIN(M) 1487 is surprising. This isolate, deposited as *Ceratocystismontium*CBS 137.36 by Rumbold in 1936 (see MycoBank: 535049 for taxonomic history), has been designated by CBS (Westerdijk Fungal Biodiversity Institute) as the authoritative (AUT) strain for *Ophiostomaips* (Rumbold) Nannf. (the original type or ex-type for *O.ips* is lost). Kim et al. (2003), based on nuclear markers and the ability of CBS 137.36 to grow at 35 °C, argued that this strain belongs to *O.ips* and can be distinguished from *O.montium*. In most fungi, mitochondria are uniparentally inherited, but on rare occasions, segments of mitochondrial genomes can move horizontally between different fungal species (see Mayers et al. 2021). The similarity of the *cob*-201 intron and the 18 nt segment in the downstream exon in the CBS 137.36 with *O.montium* may hint at a possible hybridization/introgression event between these two species (or other members of the “*ips*” lineage). Hybridization and introgression events have been recorded for members of *Ophiostomaulmi* and the subspecies of *Ophiostomanovo-ulmi* (Hessenauer et al. 2020). In future studies, more strains of *O.ips* and *O.montium* need to be examined at both the nuclear and mitogenomic levels to provide evidence for this speculation.

## ﻿Conclusion

Mitochondrial genomes among members of the *Ophiostomatales* vary in size in part due to the gain and loss of mobile introns, and introns can expand by being invaded by mobile introns. Mitochondrial genomes provide insights into the taxonomy and evolution of fungi and offer some insights into the evolutionary dynamics of mitochondrial introns, such as one example of a nested intron arrangement in strains of *O.ips* that appears to splice by a novel ratchet (also referred to as recursive splicing) splicing mechanism. As far as we know, this would be the first demonstration of such a splicing mechanism operating in nested organellar introns, and nested introns therefore offer a platform for studying alternative splicing of complex intron configurations that may impact gene expression.

## ﻿Additional information

### Use of AI

No use of AI was reported.

### Author ORCIDs

Jigeesha Mukhopadhyay https://orcid.org/0000-0003-1137-2486

Alvan Wai https://orcid.org/0009-0005-4118-5915

B. Franz Lang https://orcid.org/0000-0003-1035-5449

Georg Hausner https://orcid.org/0000-0002-6541-1148

### Data availability

The datasets supporting the conclusions of this article are included within the article and supporting documents. GenBank accession numbers for the mitogenomes and sources for the fungal cultures used in this study are listed in Table 1. RNAseq data has been deposited in NCBI under the BioProject PRJNA1143047 (SRA accession SRX25593433).

## Supplementary materials

Supplementary material 1 Mitochondrial genome maps, figure summarizing mitogenome contents, RNA models for introns folding, phylogenetic tree Data type docx https://binary.pensoft.net/file/1379548This dataset is made available under the Open Database License (http://opendatacommons.org/licenses/odbl/1.0/). The Open Database License (ODbL) is a license agreement intended to allow users to freely share, modify, and use this Dataset while maintaining this same freedom for others, provided that the original source and author(s) are credited.Jigeesha Mukhopadhyay, Alvan Wai, B. Franz Lang, Georg Hausner
