# Supplementary material for: ﻿Characterization of the mitochondrial genomes for Ophiostomaips and related taxa from various geographic origins and related species: large intron-rich genomes and complex intron arrangements
Source: IMA Fungus. 2025 Jul 22;16:e159349. doi: 10.3897/imafungus.16.159349 (PMC12308204; doi:10.3897/imafungus.16.159349)
Supplement: Supplementary material 1 — Mitochondrial genome maps, figure summarizing mitogenome contents, RNA models for introns folding, phylogenetic tree [file imafungus-16-e159349-s001.docx]

Supplementary Figures (S1 to S12) and Table S1 (A and B)


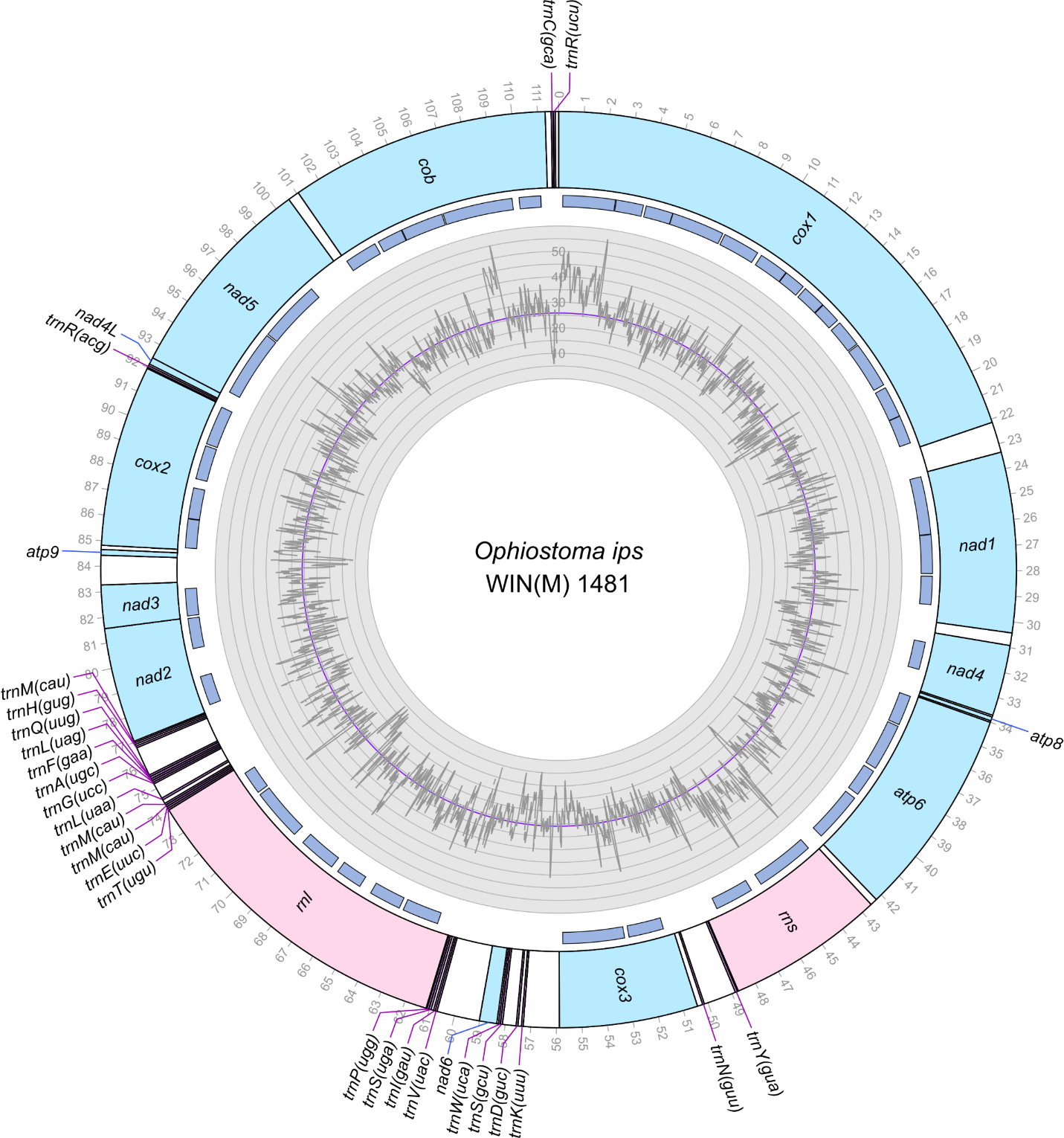


Figure S1. Circular representation of the mitochondrial genomes of *O. ips* WIN(M) 1481. Genes, introns, and GC plot are shown on the outer, middle, and inner tracks, respectively. The purple line of the GC plot corresponds to the average GC content of the mitochondrial genomes. The tick marks on the outer track label every 1,000th nucleotide, starting from the beginning of the *cox1* gene. All labeled genes are encoded on the same strand.


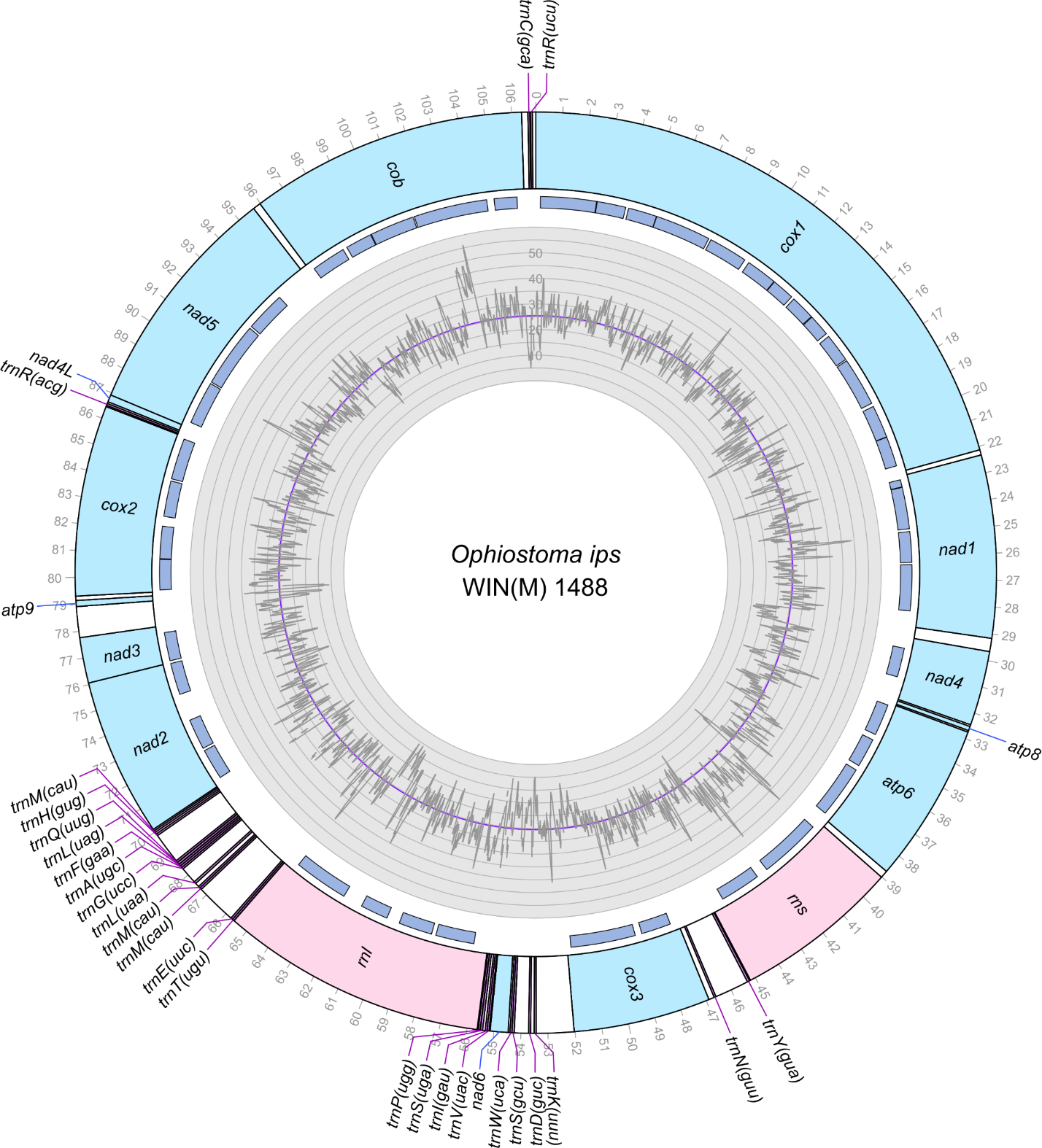


Figure S2. Circular representation of the mitochondrial genomes of *O. ips* WIN(M) 1488. Genes, introns, and GC plot are shown on the outer, middle, and inner tracks, respectively. The purple line of the GC plot corresponds to the average GC content of the mitochondrial genomes. The tick marks on the outer track label every 1,000th nucleotide, starting from the beginning of the *cox1* gene. All labeled genes are encoded on the same strand.


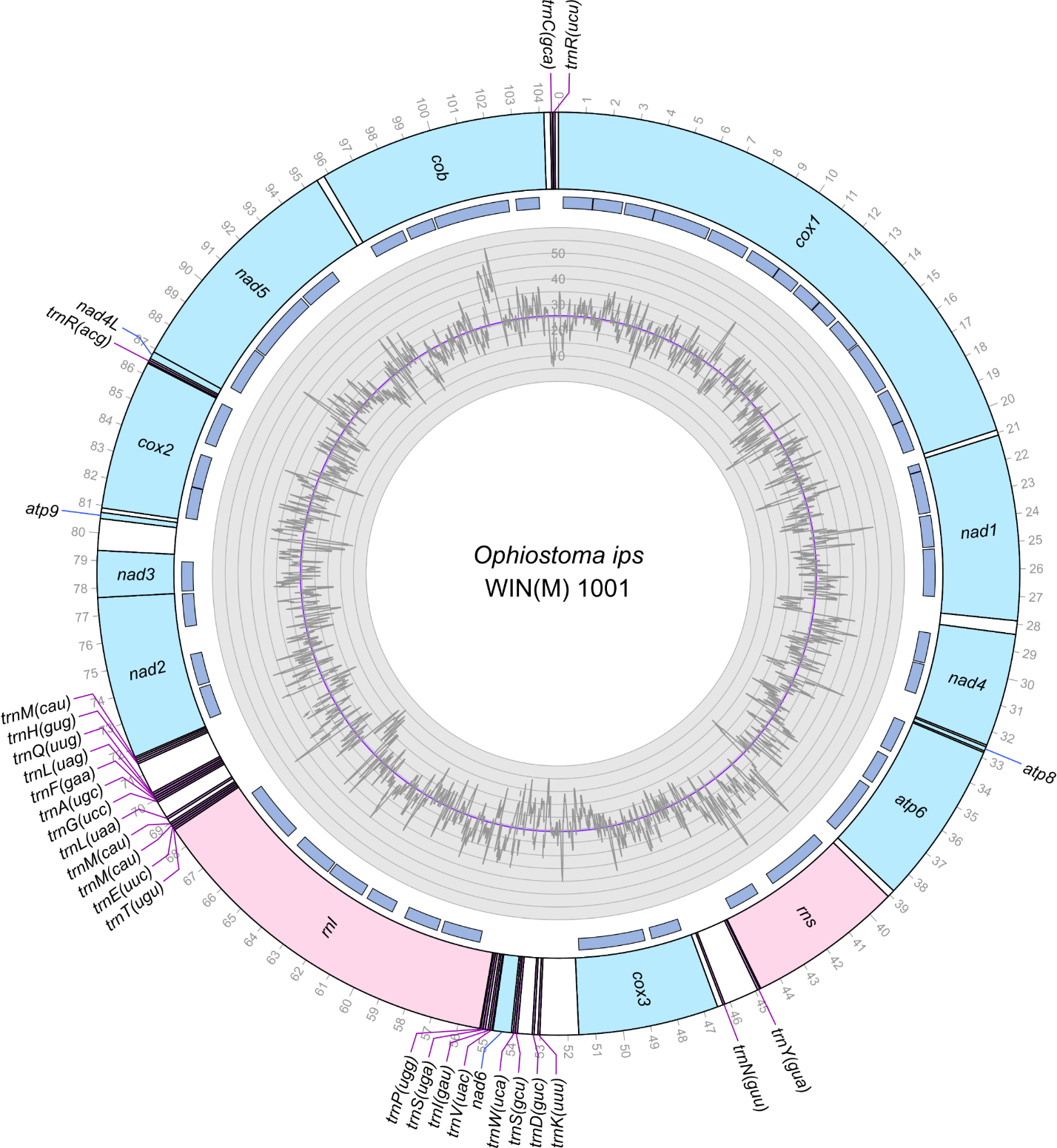


Figure S3. Circular representation of the mitochondrial genomes of *O. ips* WIN(M) 1001. Genes, introns, and GC plot are shown on the outer, middle, and inner tracks, respectively. The purple line of the GC plot corresponds to the average GC content of the mitochondrial genomes. The tick marks on the outer track label every 1,000th nucleotide, starting from the beginning of the *cox1* gene. All labeled genes are encoded on the same strand.


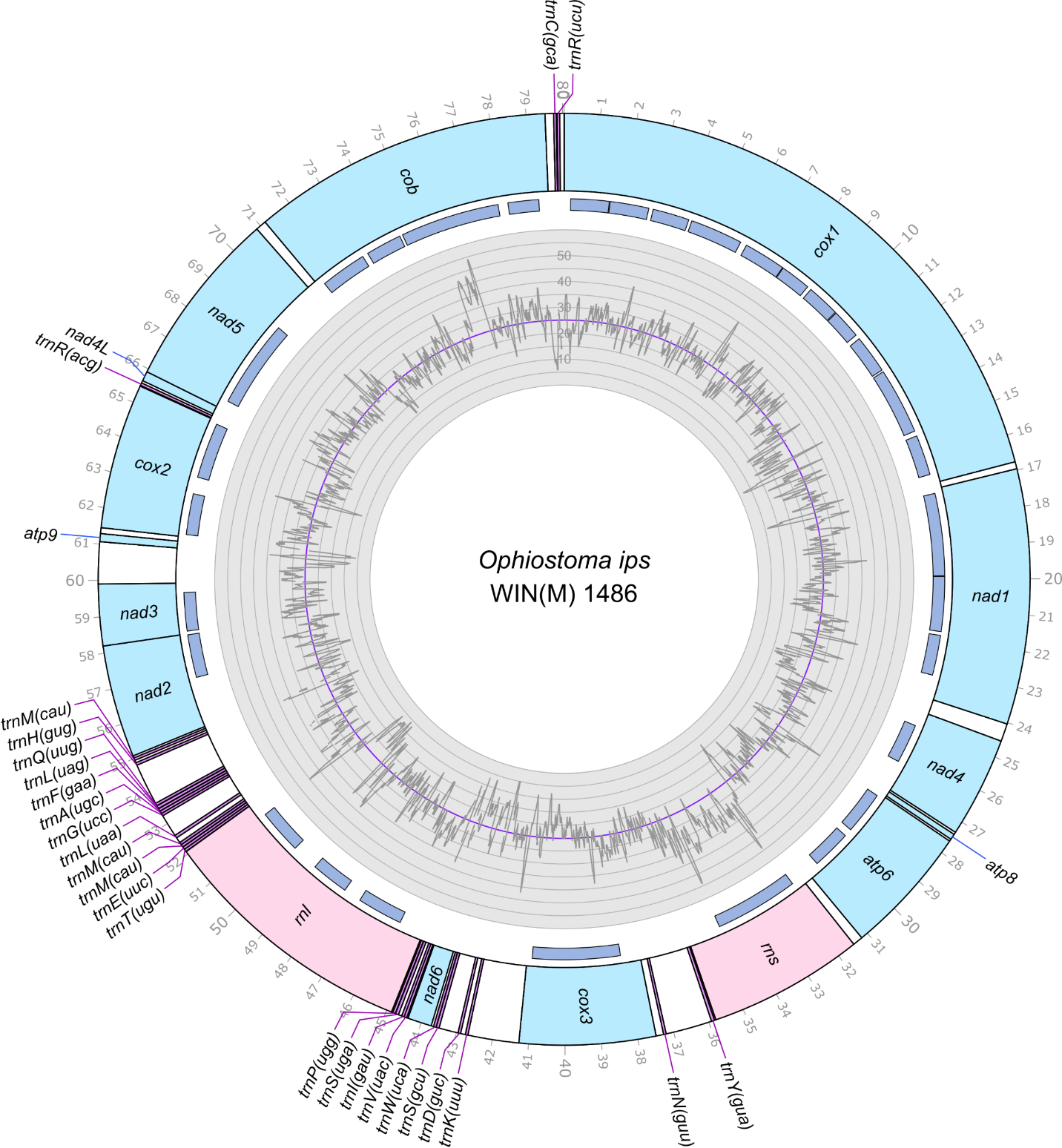


Figure S4. Circular representation of the mitochondrial genomes of *O. ips* WIN(M) 1486. Genes, introns, and GC plot are shown on the outer, middle, and inner tracks, respectively. The purple line of the GC plot corresponds to the average GC content of the mitochondrial genomes. The tick marks on the outer track label every 1,000th nucleotide, starting from the beginning of the *cox1* gene. All labeled genes are encoded on the same strand.


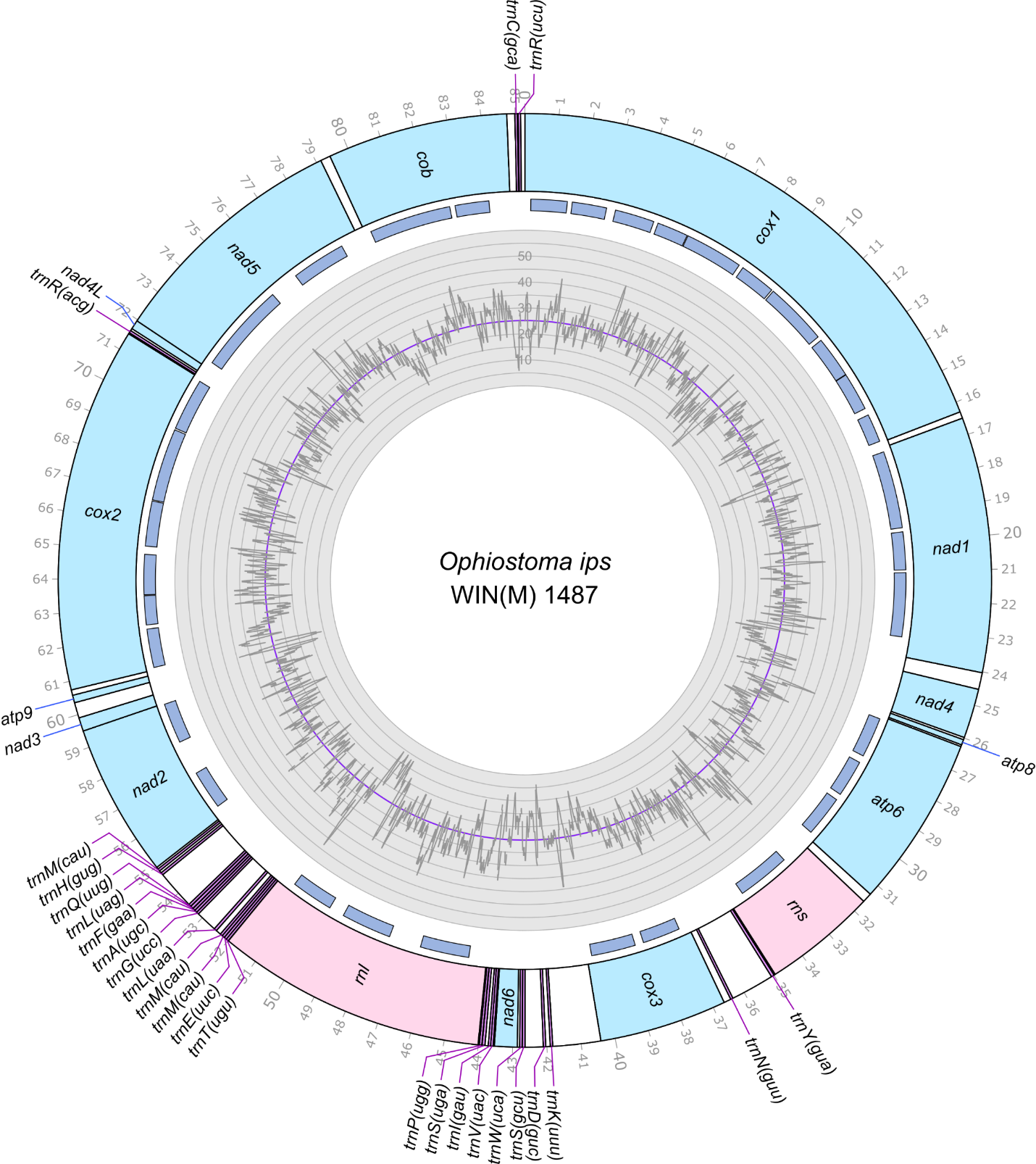


Figure S5. Circular representation of the mitochondrial genomes of *O. ips* WIN(M) 1487. Genes, introns, and GC plot are shown on the outer, middle, and inner tracks, respectively. The purple line of the GC plot corresponds to the average GC content of the mitochondrial genomes. The tick marks on the outer track label every 1,000th nucleotide, starting from the beginning of the *cox1* gene. All labeled genes are encoded on the same strand.


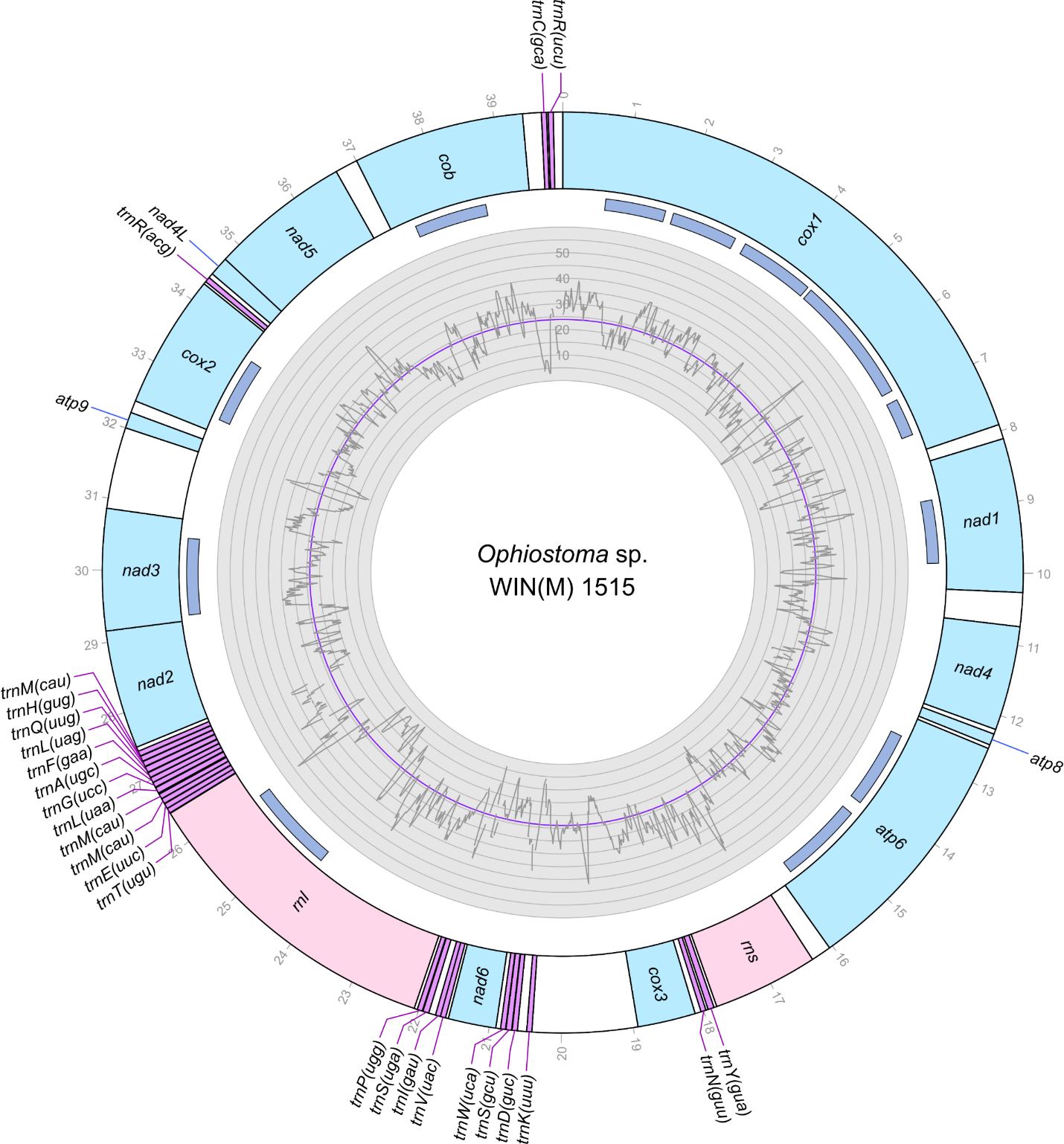


Figure S6. Circular representation of the mitochondrial genomes of *O. ips* WIN(M) 1515. Genes, introns, and GC plot are shown on the outer, middle, and inner tracks, respectively. The purple line of the GC plot corresponds to the average GC content of the mitochondrial genomes. The tick marks on the outer track label every 1,000th nucleotide, starting from the beginning of the *cox1* gene. All labeled genes are encoded on the same strand.


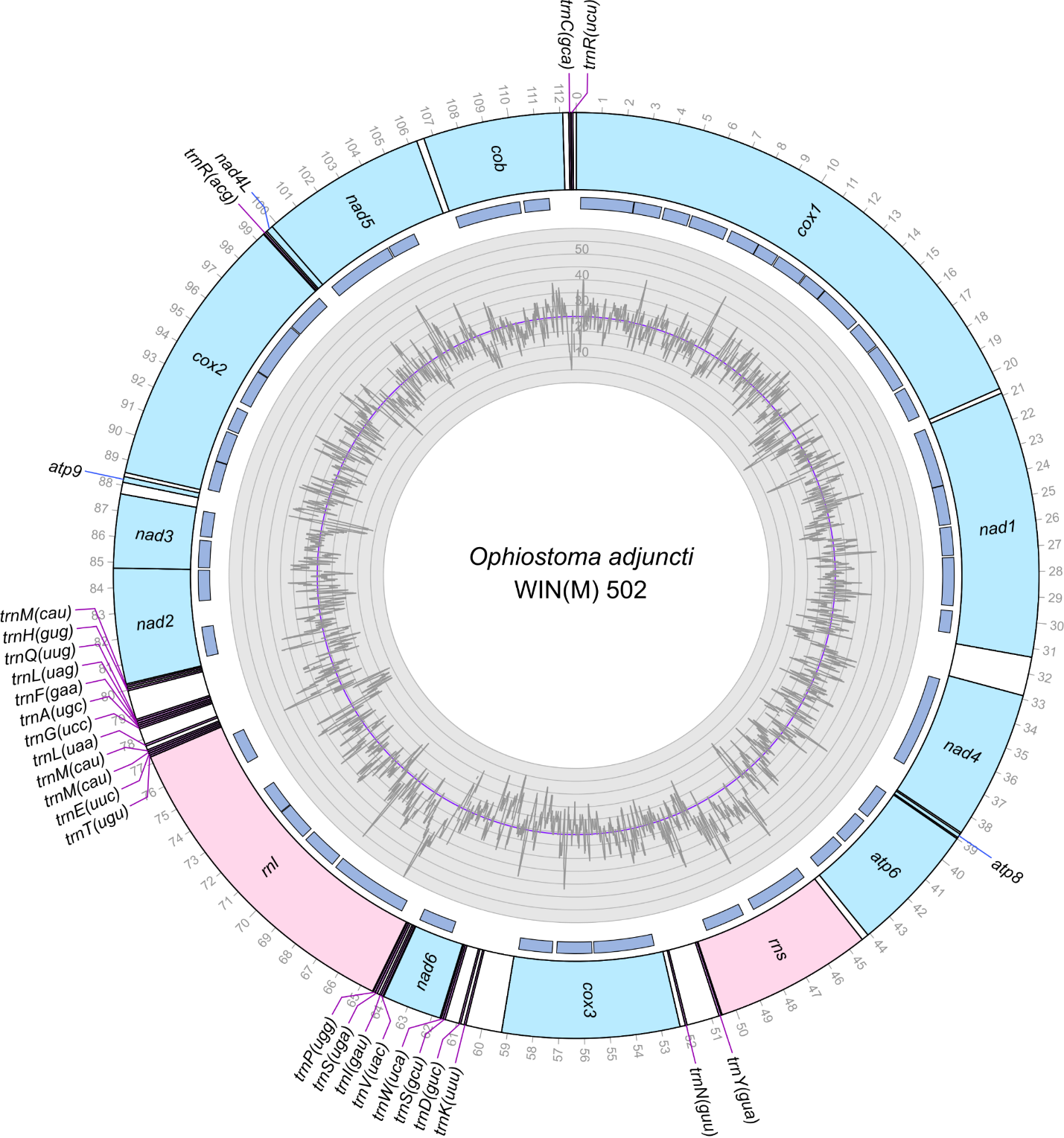


Figure S7. Circular representation of the mitochondrial genomes of *O. adjuncti* WIN(M) 502. Genes, introns, and GC plot are shown on the outer, middle, and inner tracks, respectively. The purple line of the GC plot corresponds to the average GC content of the mitochondrial genomes. The tick marks on the outer track label every 1,000th nucleotide, starting from the beginning of the *cox1* gene. All labeled genes are encoded on the same strand.


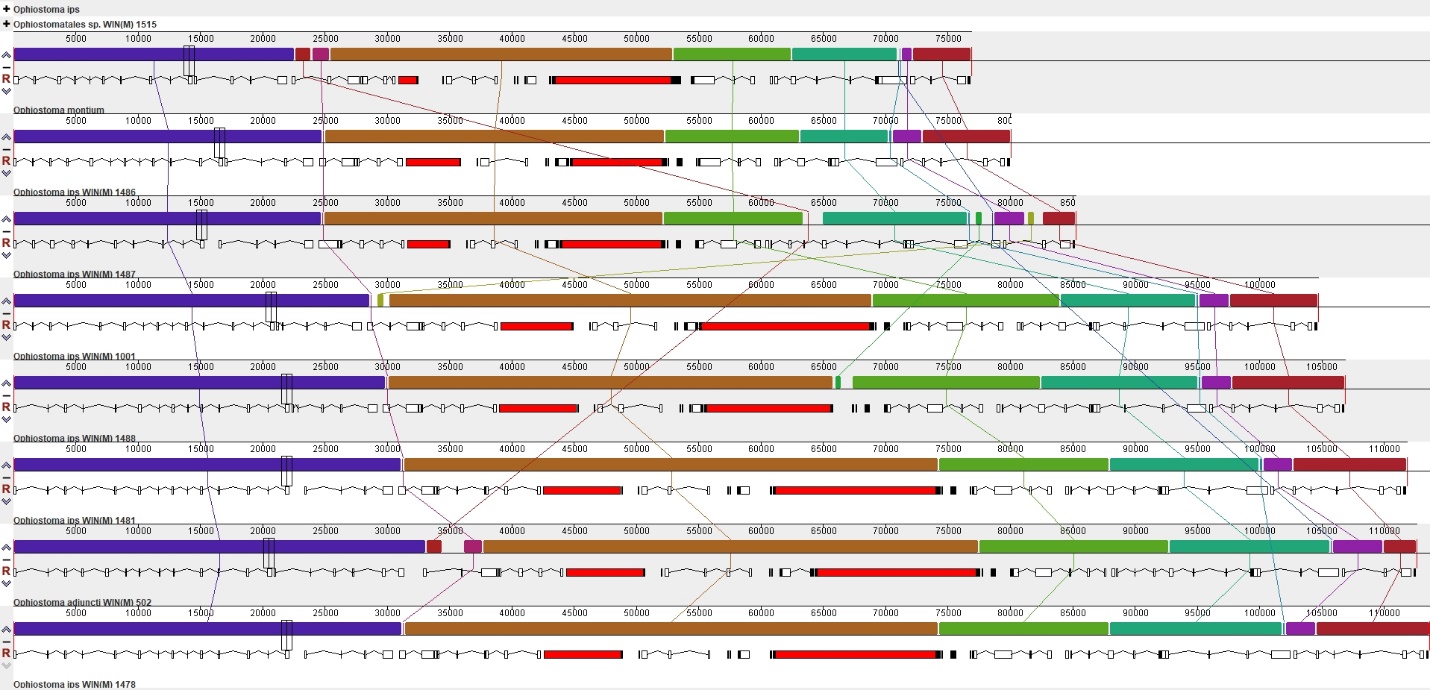


Figure S8. Mauve progressive alignment of the mitochondrial genomes for the ten members of the *O. ips* complex (*O. ips* strains, *O. ips*-like and related species). The mitogenomes are co-linear and therefore show no evidence of genome rearrangement. Lines connecting Locally Collinear Blocks (LCBs) are shown that represent conserved (homologous) segments of sequences across the genomes. WIN(M) 1515 is selected as the reference genome due to the smallest size and least number of introns among the studied species. The other species with correspondingly larger genomes and huge number of introns can be viewed as evidence of intron gain/loss events with respect to WIN(M) 1515.


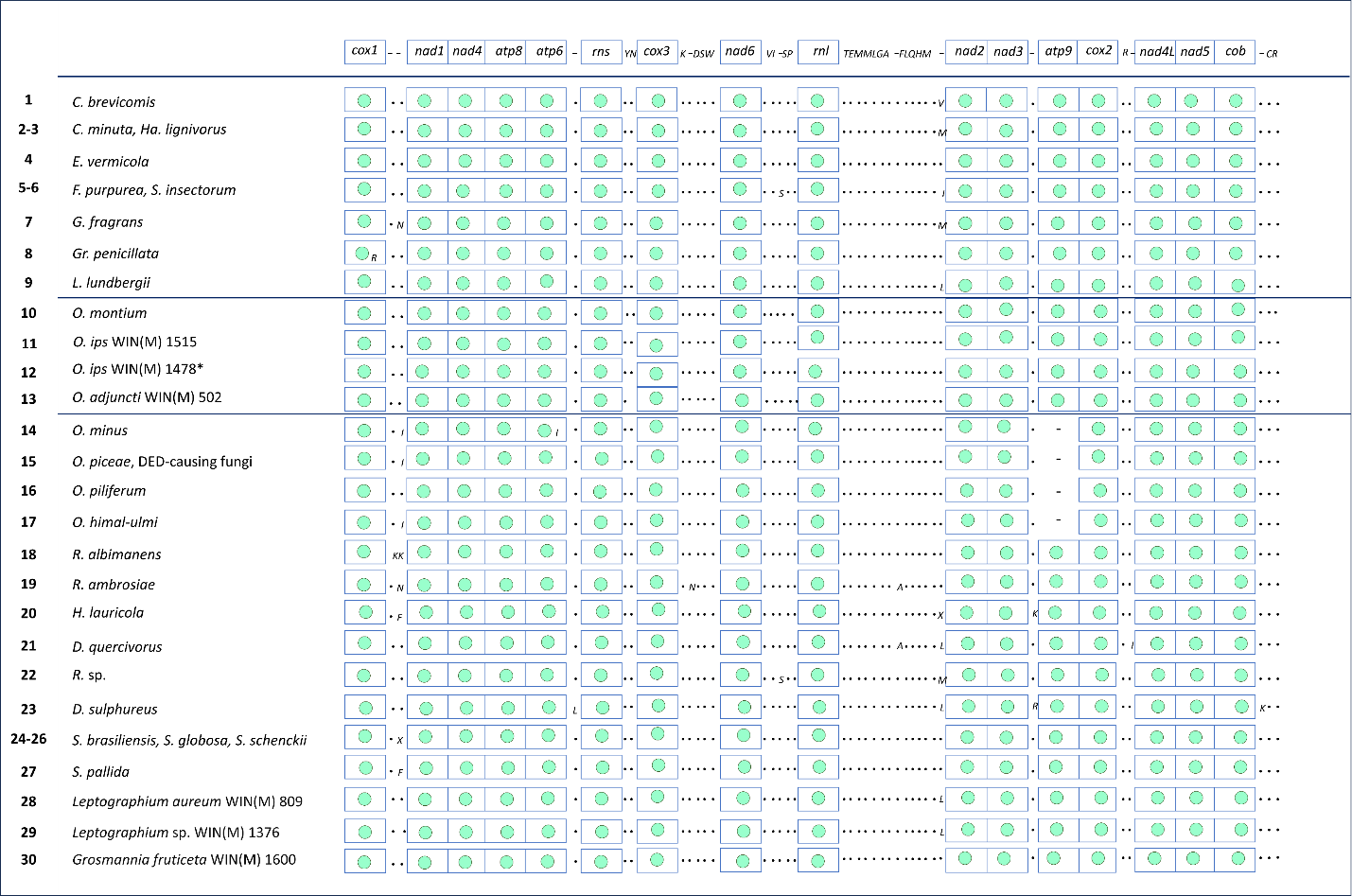


Figure S9. Gene synteny for 37 members of the Ophiostomatales. Amino acids are represented with the single-letter code. C., *Ceratocystiopsis*; D., *Dryadomyces*; E., *Esteya*; F., *Fragosphaeria*; G., *Graphilbum*; Gr., *Grosmannia*; H., *Harringtonia*; Ha., *Hawksworthiomyces*; L., *Leptographium*; O., *Ophiostoma*; R., *Raffaelea*; S., *Sporothrix* ; N/A, not applicable; –, absence of gene; ·, presence of genes. * Indicates that the *O. ips* strains WIN(M) 1478, 1480, 1481, 1486, 1487, 1488, 1001, and the *ips*-like *Ophiostoma* sp. WIN(M) 1515 are represented by *O. ips* WIN(M) 1478 and they shared identical gene synteny. Related species *O. montium* and *O. adjuncti* also share the gene order. The *O. ips* strain NTMB01000349.1 seemed to be missing the *atp8* gene containing segment in the scaffold_143 (Zubaer *et al.* 2021).


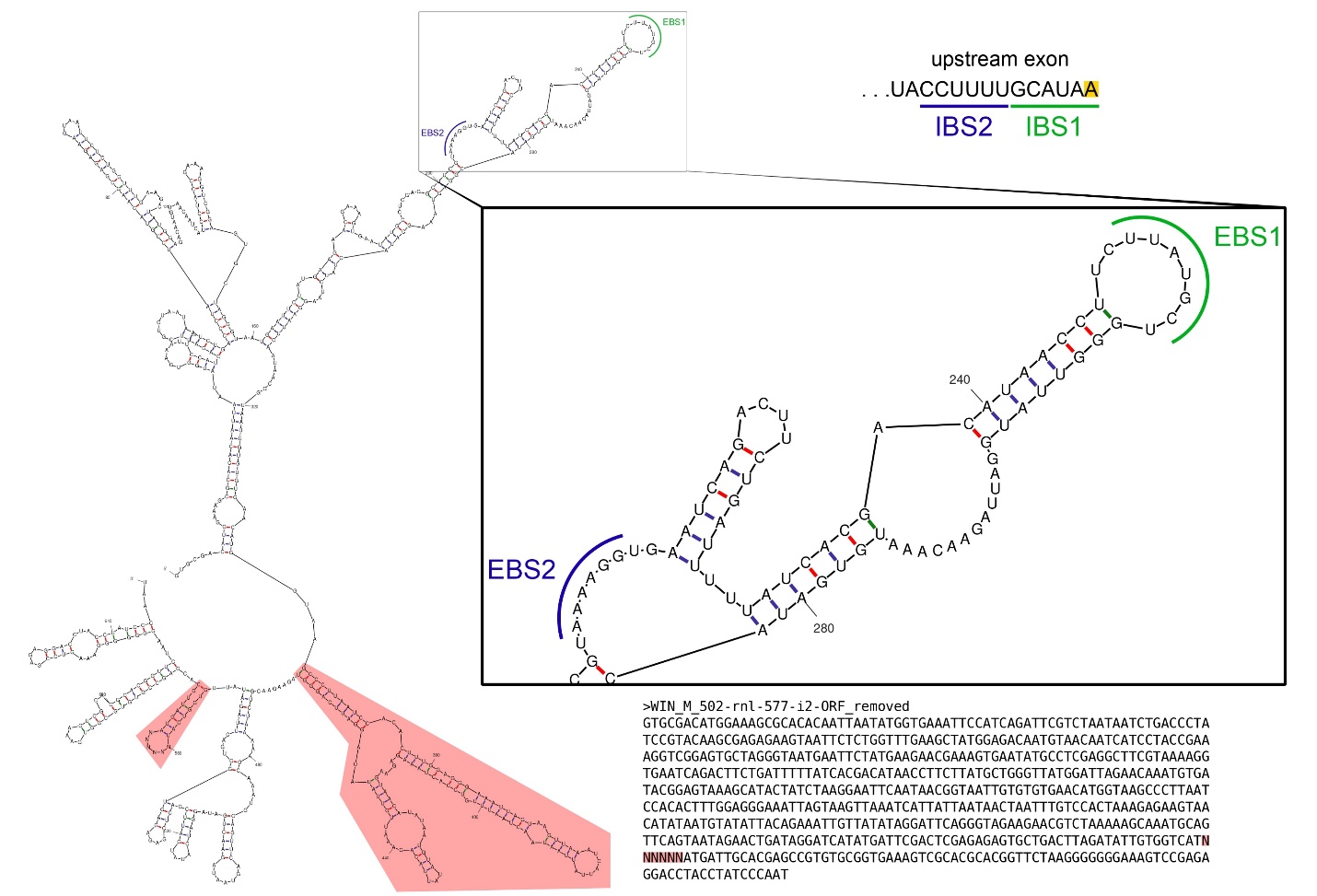


Figure S10. mfold model of *rnl*-576 in WIN(M) 502. Domains II and IV are highlighted in red. It is indicated that *rnl*-576 does not start or end with typical, conserved nucleotides.


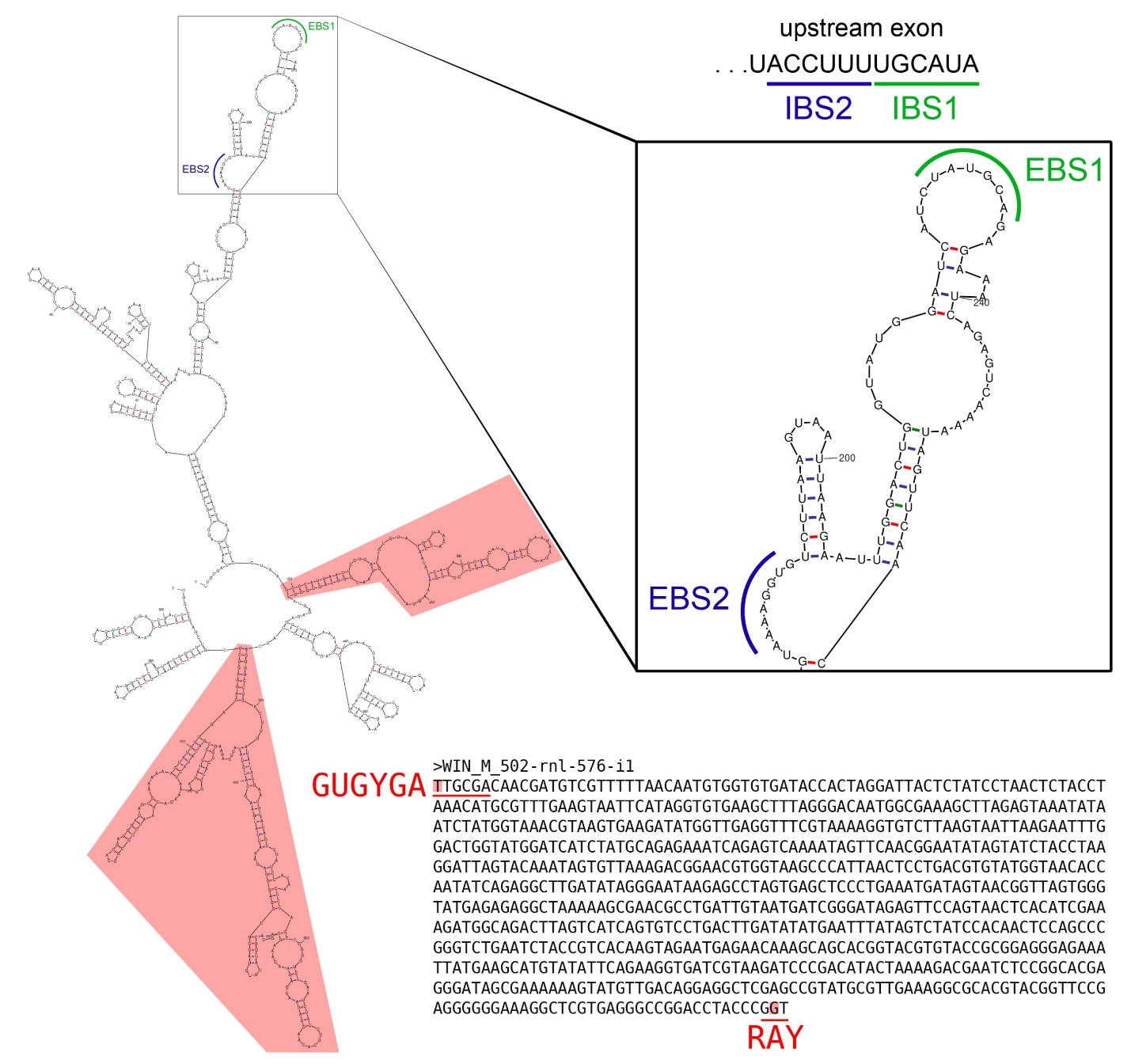


Figure S11. mfold model of *rnl*-577 in WIN(M) 502. Domains II and IV are highlighted in red. It is indicated that for domain IV, a region coding for a RT was removed and replaced by six Ns.


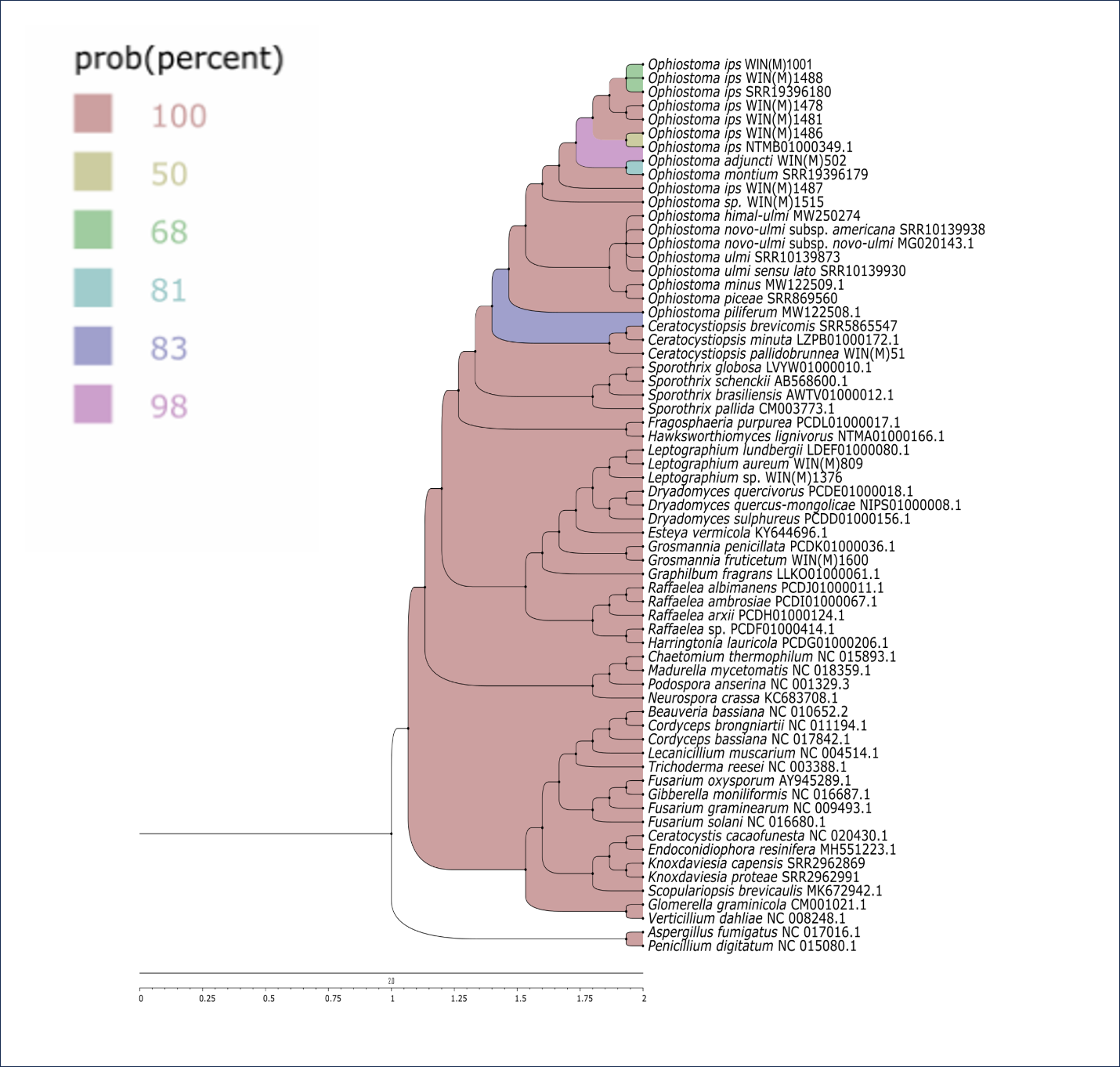


Figure S12. Phylogenetic relationships of 65 fungal species belonging to the Ascomycota, including 43 members of the Ophiostomatales are presented, based on concatenated amino acid sequences. *Aspergillus fumigatus* and *Penicillium digitatum* are selected as the outgroups. The IQ-TREE2 was constructed based on 1,000 bootstraps, using the best-fit model JTT (+I+G); the percentage probabilities at the nodes were grouped by representative colors. Branch lengths are proportional to the number of substitutions per site (see scale bar).

Table S1A. *cob-*490 RT-PCR primers for WIN(M) 1478 and 1480

| Primer name | Orientation | Primer Sequence 5’-3’ |
| --- | --- | --- |
| RTCOB-7F | Forward | GACATAGTCTAATTATATTTG |
| RTCOB-11Fgp2 | Forward | GCGTAAGCGAAGATGTGG |
| RTCOB-6 | Forward | CGCGTTATAGTAAATTATGC |
| RTCOB-8R | Reverse | CAAATATAATTAGACTATGTC |

Table S2. *cox3-*640 RT-PCR primers for WIN(M) 1478 and 1480

| Primer name | Orientation | Primer Sequence 5’-3’ |
| --- | --- | --- |
| Ipscox3I2-F1 | Forward | CAAGAAGTTGAATATGATC |
| Ipscox3I2-R1 | Forward | CAGTATGCAATAGCACCTTC |
| Ipscox3I2-F2 | Forward | CCAGCAGTATGAGGAGGATTAG |
| Ipscox3I2-R2 | Reverse | CCAGCAGTATGAGGAGGATTAG |
